# A predictive framework for evaluating the thermophysical properties of multi-walled carbon nanotube nanofluids dispersed in a water–ethylene glycol 50:50 base fluid

**DOI:** 10.1038/s41598-026-54075-1

**Published:** 2026-05-20

**Authors:** Ali B. M. Ali, Mahmoud Fadhel Idan, Muthanna K. Kareem, Narinderjit Singh Sawaran Singh, Abdalmalik N. Attallah, Hakim AL Garalleh, Abdulkareem Afandi, Mahmut Taner, Soheil Salahshour, Laleh Hoseini

**Affiliations:** 1 Advanced Technical College, University of Warith Al-Anbiyaa, Karbala, Iraq; 2 Department of Power Mechanical Engineering Techniques, College of Engineering Technologies, University of Al Maarif, Al Anbar, 31001 Iraq; 3 College of Engineering, Al-Ayen University, Thi-Qar, Iraq; 4 Faculty of Data Science and Information Technology, INTI International University, Persiaran Perdana BBN, Putra Nilai, Nilai, 71800 Malaysia; 5 Department of Mathematical Science, College of Engineering, University of Business and Technology, Jeddah, 21361 Saudi Arabia; 6 Faculty of Engineering and Architecture, Istanbul Gelisim University, Istanbul, Türkiye; 7 Faculty of Engineering and Natural Sciences, Istanbul Okan University, Istanbul, Turkey; 8 Faculty of Engineering and Natural Sciences, Bahcesehir University, Istanbul, Turkey; 9 Research Center of Applied Mathematics, Khazar University, Baku, Azerbaijan; 10 Faculty of Science and Letters, Piri Reis University, Tuzla, Istanbul, Turkey; 11 Fast Computing Center, Shabihsazan Ati Pars, Tehran, Iran

**Keywords:** Artificial neural networks, Temperature, Volume concentrations, Base fluid, Energy efficiency, Engineering, Materials science, Mathematics and computing, Nanoscience and technology

## Abstract

This study presented a predictive optimization framework for evaluating the thermophysical properties of multi-walled carbon nanotube NFs dispersed in a 50:50 water–ethylene glycol base fluid. The main objective was to simultaneously predict TC and dynamic µ_nf_, addressing a key limitation of previous studies that focused primarily on TC alone. A feedforward artificial neural network with two hidden layers was developed and validated using experimental data. The dataset covered nanoparticle volume concentrations between 0.025% and 0.1% and temperatures ranging from 25 °C to 80 °C. The proposed model demonstrated strong predictive capability across all evaluation metrics. Under 10-fold cross-validation, the root mean square error for TC varied from 1.31 × 10⁻⁴ to 3.71 × 10⁻⁴ W/m·°C, while the corresponding values for µ_nf_ range from 0.010 to 0.031 mPa·s. Low mean-squared error values across the training, validation, and test datasets confirmed the robustness of the learning process. Optimal performance was achieved at epoch 5 for TC and at epoch 8 for µ_nf_. In all cases, the coefficient of determination exceeded 0.99, indicating excellent agreement between predictions and experimental measurements. Relative errors remained limited to 0.32–1.57% for TC and 0.12–0.25% for µ_nf_, while absolute errors were also tightly bounded. A complementary sensitivity analysis further supported the model stability. A 10% variation in Temperature led to maximum deviations of 2.636% in TC and 0.623% in µ_nf_, whereas the same variation in nanoparticle concentration produced larger deviations of 5.744% and 0.893%, respectively. Despite this difference, mean deviations remained modest for both properties, confirming the robustness of the proposed framework under input perturbations.

## Introduction

 The continuous increase in global energy demand, along with rising costs and environmental concerns, has underscored the urgent need to improve energy efficiency and develop effective thermal management strategies in modern engineering systems. Heating and cooling processes account for a considerable share of overall energy consumption, and their optimization not only reduces operational costs but also mitigates greenhouse gas emissions and enhances sustainability. Conventional heat transfer (HT) fluids, such as H₂O, ethylene glycol (EG), and mineral oils, have been widely used due to their abundance, non-toxic nature, and low cost. However, their intrinsically low thermal Conductivity (TC) significantly restricts their performance in high-heat-flux applications. This fundamental limitation has driven the development of advanced fluids with superior thermophysical properties. In this context, nanofluids (NFs), defined as suspensions of nanoparticles (NPs) in base fluids, were identified as promising candidates^1–5^.The incorporation of NPs, such as Al₂O₃, TiO₂, CuO, SiO₂, and SiC, can enhance TC by creating additional conduction pathways, intensifying Brownian motion, and generating interfacial layers with high thermal transport capacity^6^. Numerical simulations and theoretical models further indicate that particle type, size, shape, and concentration, along with the properties of the base fluid, critically affect both the thermal and rheological behavior of NFs^7^. Investigations on carbon-based and metallic NFs demonstrated simultaneous improvements in TC and colloidal stability^8^. Moreover, a variety of base fluids, including deionized water (H_2_O), EG, alcohols, oils, and refrigerants, were explored for NF formulations^9^. Data-driven approaches, such as artificial neural networks (ANNs), were applied to predict viscosity (µ_nf_) and TC with high accuracy^10^. Practical applications of NFs have already been reported in natural convection enhancement^11^ and compact heat exchangers^12^.Despite these advances, issues, such as increased µ_nf_, colloidal instability, and high preparation costs, continue to limit the widespread use of NFs^13^. To address these challenges, HNFs, which contain two or more types of NPs within a single base fluid, were developed^14^. HNFs demonstrated superior performance in renewable energy systems, with solar collectors achieving efficiency gains of over 25%. Additionally, they showed effectiveness in thermal energy storage, where hybrid additives accelerate melting and solidification, and in electronic cooling, where hotspots and thermal resistance were effectively reduced. These advances establish HNFs as versatile and promising candidates for next-generation thermal management technologies^15^. Recent investigations have increasingly focused on HNFs and their thermophysical properties, with particular emphasis on experimental characterization and predictive modeling using machine learning (ML) approaches, such as ANNs. Çolak et al.^16^ examined the TC of Al₂O₃–Cu/HNFs over a volume concentration (φ) range of 0.0125–0.2% and temperatures (T) between 10 and 65 °C. Their ANN-based model achieved an average deviation of only 0.4%, while Maxwell’s classical correlation showed the closest agreement with experiments, with a deviation of 0.08%. Similarly, Ruhani et al.^17^ reported that for CeO₂/EG-NFs, the TC ratio increased monotonically with both NP loading (0–2.5%) and T (25–50 °C). Their ANN model with seven neurons provided nearly perfect agreement with experiments (*R* ≈ 1), confirming the robustness of ANN in low-dimensional problems. Expanding this approach, Yang et al.^18^ developed ternary HNFs consisting of multi-walled carbon nanotubes (MWCNTs), TiO₂, and ZnO dispersed in H₂O–EG (80:20). For φs of 0.1–0.4% and Ts of 25–50 °C, their ANN with 26 neurons reproduced 102 experimental data points with a maximum absolute error below 0.018, further demonstrating the scalability of ANN architectures to more complex fluid systems. Beyond TC, other studies investigated system-level applications. Chilambarasan et al.^19^ evaluated Al₂O₃–H_2_O-EG-NFs in solar flat plate collectors using NP-φ of 0.01–0.2% and flow rates of 0.024–0.048 kg/s. At 0.2% loading and 0.036 kg/s, efficiency improved by 54.1% relative to the base fluid, and ANN models accurately reproduced the system’s dynamic thermal response, highlighting the applicability of ML beyond property estimation. Giwa et al.^20^ focused on alumina–ferrofluids [Al₂O₃–Fe₂O₃ (25:75 mass%)] dispersed in both DIW and EG–DIW mixtures under 20–50 °C and φ = 0.05–0.75%. They reported substantial µ_nf_ increases (up to 43.64%) and electrical conductivity enhancements exceeding 7600%, with both the ANN and adaptive neuro-fuzzy inference system (ANFIS) models achieving errors below 4.5% and 3.9%, respectively, significantly outperforming conventional regression-based correlations. Although significant progress was made in the field of HNFs, important gaps remain. Tota et al.^21^ addressed the limitations of single-component NFs by investigating hybrid Al₂O₃/MWCNT-NFs as an effective route to simultaneously enhance TC and HT performance in passive systems. Their results demonstrated that, at a low NP loading of 0.028 vol% and a Reynolds number of approximately 3093, the use of HNFs in conjunction with passive geometric modifications led to a pronounced increase in the thermal performance factor, indicating a favorable balance between HT enhancement and flow resistance. In addition, data-driven analysis revealed that advanced machine learning models can accurately capture the nonlinear relationship between operating conditions and thermal performance, with prediction accuracies reaching R² ≈ 0.99 for the best-performing model. Haldar et al.^22^ examined the TC enhancement of hydraulic oil–based hybrid nano-lubricants to improve HT capability in engineering systems. Their results demonstrate that TC showed a strong and nonlinear dependence on both NP- φ and T, reaching a maximum enhancement of about 62.9% at elevated loadings. The study further showed that surrogate modeling approaches could accurately represent this behavior, with the proposed response surface correlation achieving R² ≈ 0.999, while the ANN also provided reliable estimates (R² ≈ 0.986). Alqaed et al.^23^ examined the stability and TC characteristics of H_2_O/CeO₂–MWCNT-HNFs to clarify their applicability in HT and thermal energy storage systems. Their results indicate that the synergistic interaction between CeO₂ NPs and MWCNTs led to a stable suspension and a marked enhancement in TC, which increased consistently with both solid φ and T. The proposed correlation yielded a coefficient of determination (R) of 0.992, while the ANN achieved comparably high predictive accuracy, with a maximum deviation of 0.5%. Ganguly et al.^24^developed an MXene-based magnetic nanohybrid coating on a cellulose microporous membrane for flexible electronic applications using a vacuum-assisted filtration strategy. Their investigation demonstrated that the incorporation of photopolymerized polynorepinepherene-coated MXene and iron oxide NPs significantly enhanced the mechanical flexibility, cyclic deformation tolerance, solvent resistance, and electromagnetic interference shielding performance of the membrane. Esawi et al.^25^investigated the effect of CNT reinforcement on the mechanical performance of aluminum-based composites prepared through ball milling, cold compaction, and hot extrusion techniques. Their results demonstrated that uniform CNT dispersion significantly improved the tensile strength and stiffness of the Al matrix, with enhancements of approximately 50% and 23%, respectively, compared to pure aluminum.Overall, recent experimental and data-driven investigations demonstrated that machine-learning-based models can effectively capture the nonlinear thermophysical behavior of MWCNT-based and HNFs, including systems formulated in H_2_O–EG mixtures. Nevertheless, most existing studies relied predominantly on purely data-driven formulations, with limited integration of physics-informed interpretation or uncertainty quantification, and rarely provided a unified assessment of TC and µ_nf_ within a bounded, design-relevant operating domain. Despite the growing body of literature on NFs and HNFs, many studies examined only narrow combinations of NP types and operating conditions, thereby limiting the transferability of their conclusions to real engineering systems. Moreover, prior research predominantly emphasized TC enhancement, while the µ_nf_, which directly governs pumping power, flow resistance, and overall system efficiency, received comparatively less systematic attention. Since thermal performance improvements were meaningful only when evaluated alongside the associated µ_nf_ penalty, a coupled assessment of both properties was essential for advancing NF-based thermal management technologies. To address these gaps, the present study developed a deployment-oriented, data-driven predictive framework for the thermophysical behavior of MWCNT-NFs dispersed in a H₂O–EG, 50:50 base fluid. The analysis spanned a design-relevant operating domain, with NP-φ between 0.025 and φ = 0.1% and T = 25 to 80 °C. A feedforward ANN was employed to predict both TC and µ_nf_, with model robustness ensured through repeated cross-validation, learning-curve convergence analysis, and explicit definition of the validity domain. Beyond pointwise prediction accuracy, the proposed framework incorporated uncertainty-aware modeling through ensemble-based prediction intervals and experimental uncertainty propagation. In addition, a global, interaction-aware sensitivity analysis was conducted to elucidate the relative and coupled influence of T and NP loading, providing physically interpretable insight into the competing mechanisms governing conductivity enhancement and µ_nf_ increase. The resulting framework established a practical pathway for balancing TC gains against µ_nf_ penalties and for translating experimental NF data into actionable design guidelines for advanced thermal management and HT systems. *In the present study*,* the term “optimization” referred to the identification of favorable thermo-hydraulic operating conditions through the coupled predictive assessment of TC and dynamic µ*_*nf*_, *rather than the implementation of a formal mathematical optimization algorithm. The proposed methodology therefore emphasized data-driven performance evaluation and operating-condition assessment within the investigated design space.*

## Train/test methodology

**Fig. 1:**
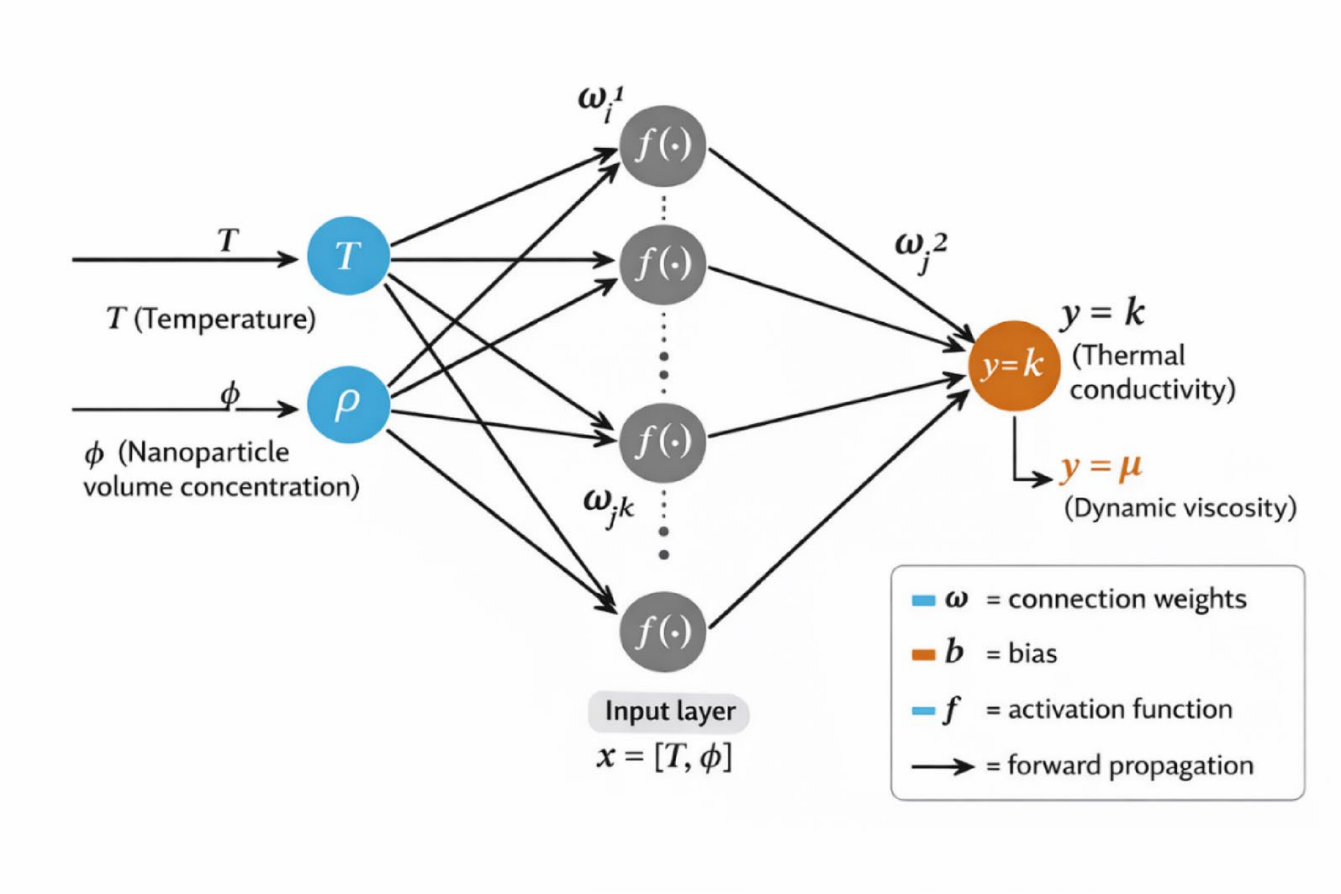
Architecture of a two-layer backpropagation neural network.

In this study, ANNs were developed using a feedforward topology, one of the most established frameworks in supervised learning. In this configuration as shown in Fig. 1, information propagated unidirectionally from the input layer through the hidden layers to the output layer, without any recurrent or feedback connections^26^. This structure contrasted with recurrent neural networks (RNNs), where internal feedback allowed temporal dependencies between outputs and subsequent states^27^. Each hidden neuron computed a weighted sum of its inputs and applied a nonlinear transfer function, enabling the network to capture complex, nonlinear interactions within the dataset^28,29^. Successive transformations across hidden layers generated increasingly abstract feature representations, allowing the model to approximate highly nonlinear input–output relationships. The base fluid (H_2_O–EG, 50:50) was considered the reference medium, with two key operating parameters selected as inputs: T and NP-φ. T varied from 25 to 80 °C, while the φ ranged between 0.025 and 0.1%. Two separate ANN models were constructed to predict TC and µ_nf_. Each network consisted of two hidden layers with 10 neurons, employing a tangent sigmoid activation function in the hidden layers to capture nonlinearities and a linear activation function in the output layer to accommodate continuous property values^30^. A total of 30 experimental data points were used for each property^31^. The dataset was randomly partitioned into 60% for training, 20% for validation, and 20% for testing. This partitioning ensured a balanced distribution across all subsets and provided representative coverage of the data domain. Before training, all inputs and outputs were normalized to the [0,1] range to ensure consistent scaling and enhance model stability. Normalization minimized the risk of dominant variables biasing the learning process, promoted faster convergence, and improved generalization to unseen data. Training incorporated an early stopping mechanism that terminated optimization if validation error failed to improve for 6 consecutive iterations, thereby reducing the risk of overfitting. The final network configuration, including the number of hidden neurons, activation functions, and optimization strategy, was determined through iterative testing and refinement to achieve stable and accurate predictive performance. The decision to employ two separate single-task neural networks, rather than a multi-task architecture with shared layers, was motivated by the distinct physical behavior and sensitivity of TC and µ_nf_ with respect to T and φ. These properties showed different error scales and monotonic trends, and enforcing a shared internal representation may compromise numerical stability or physical consistency. The adopted single-task formulation ensured stable convergence and preserved physically meaningful input–output relationships for each property (Fig. 1).

**Fig. 2:**
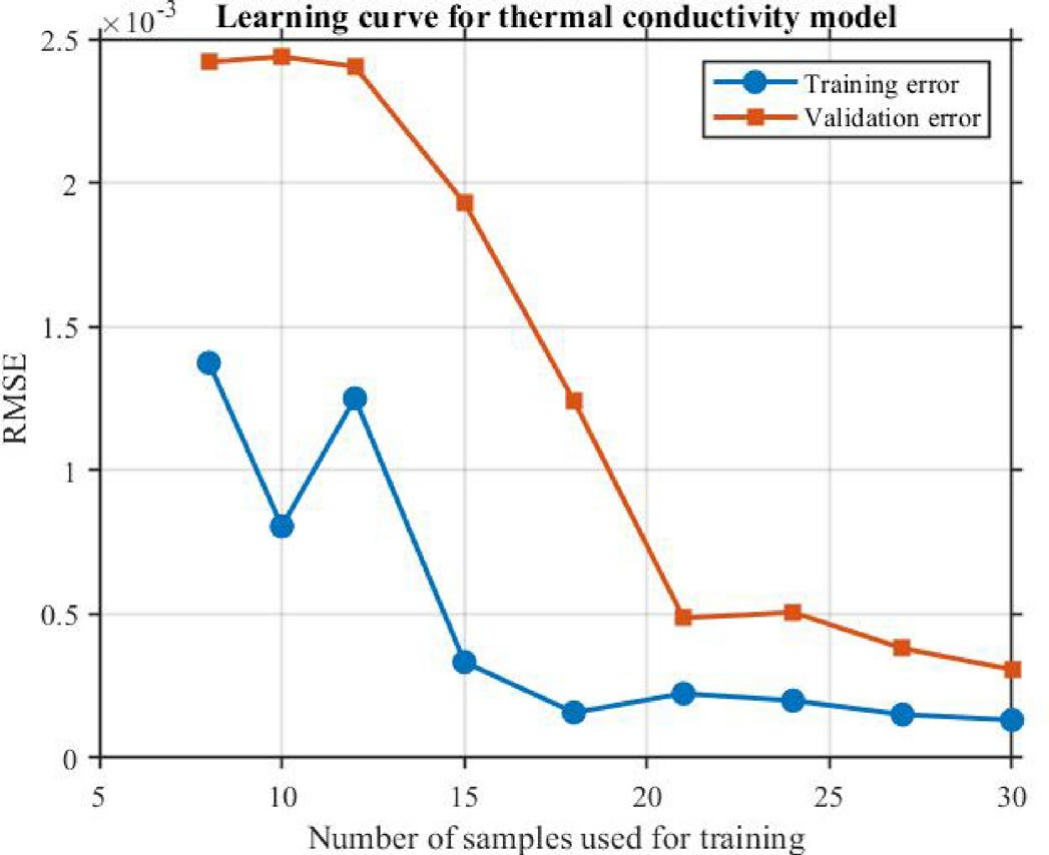
Learning curve of the ANN model showing convergence of training and validation RMSE with increasing training-set size.

The adequacy of a limited experimental dataset for data-driven modeling depended on the dimensionality of the input space and the smoothness of the underlying physical relationships. In the present framework, the mapping between thermophysical properties and the governing variables was defined over a low-dimensional domain and exhibited continuous, monotonic behavior within the investigated T and φ ranges. Under such conditions, learning-curve analysis (Fig. 2) indicated that predictive performance reached a stable regime as the training-set size increased, with validation error converging beyond approximately 20 samples. This convergence showed diminishing sensitivity to additional data and implied that the available sampling density was sufficient to resolve the functional dependence within the bounded parameter space. The consistently small separation between training and validation errors further indicated that the high R² > 0.99 arose from stable interpolation supported by adequate experimental coverage, rather than from overfitting or undersampling effects. Accordingly, the dataset was representative for predictive modeling and optimization within the defined operating domain, while extrapolative use beyond these bounds remained outside the scope of the present framework.

### Validity domain and out-of-distribution behavior of the predictive model

**Fig. 3:**
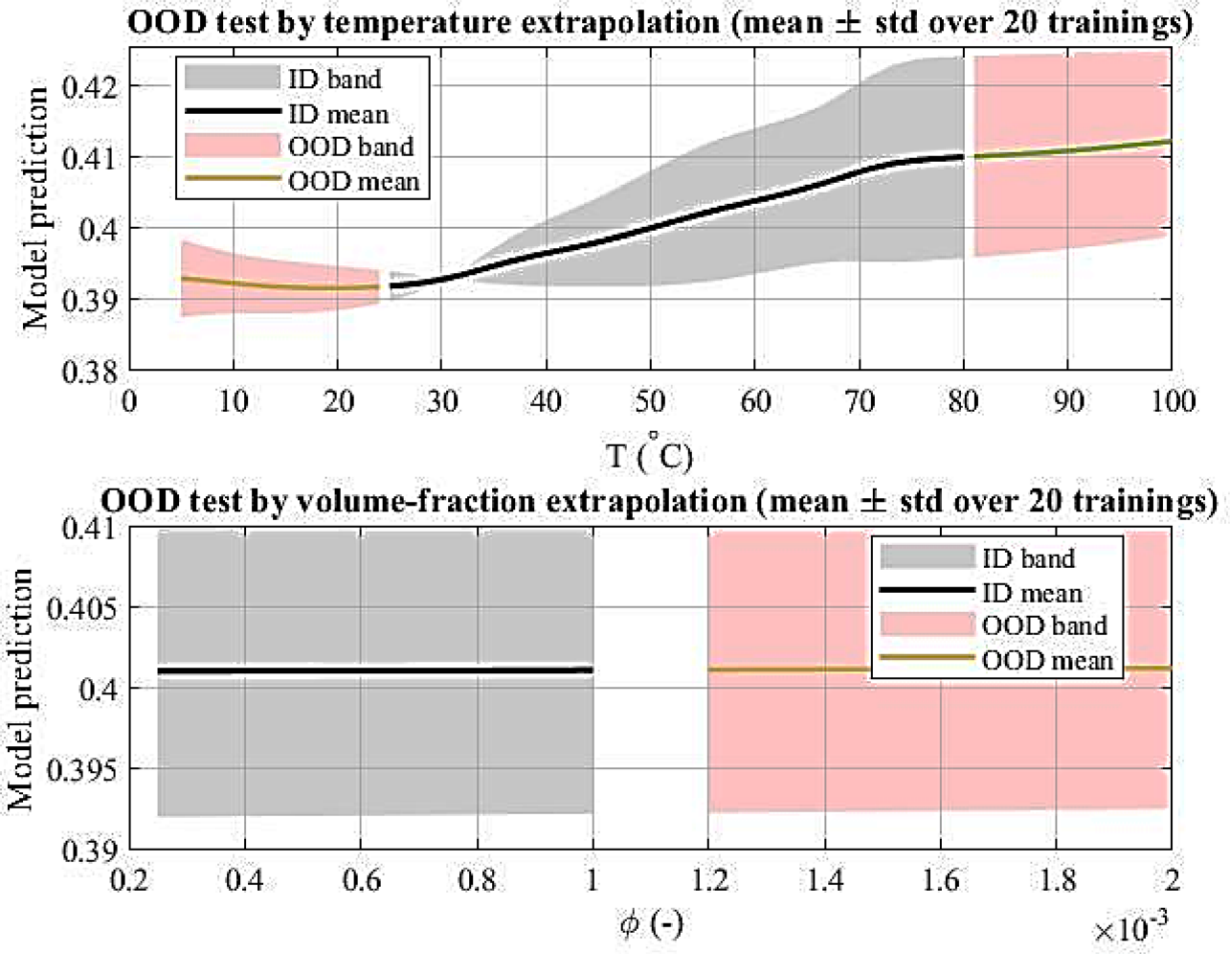
OOD behavior of the model under T and φ extrapolation.

The proposed predictive framework was inherently data-driven; therefore, its reliability was confined to the domain covered by the training dataset. In the present study, the model’s applicability was explicitly limited to T = 25–80 °C and φ < 0.1%. The base fluid was fixed as an H_2_O–EG mixture with a 50:50 mass ratio, and the particle morphology (MWCNT size and aspect ratio) matched the experimental conditions used for model development. Consequently, model predictions were considered valid only within these predefined bounds. To examine model behavior outside the training manifold, an out-of-distribution (OOD) analysis was performed by extrapolating the inputs beyond the validated T and φ ranges and evaluating prediction stability across multiple independent network trainings. For T extrapolation, the mean standard deviation of predictions within the in-distribution (ID) region was approximately 7.95 × 10^−3^, while the corresponding value under OOD conditions increased to 8.77 × 10^−3^. Similarly, for φ extrapolation, the mean prediction standard deviation within the ID domain was 8.94 × 10^−3^, compared with 8.76 × 10^−3^ for OOD inputs (Fig. 3).

These results indicate that extrapolative queries did not induce catastrophic numerical divergence; however, prediction variability outside the training domain was comparable to, and in some cases slightly higher than, that observed within the interpolation regime. This behavior showed a transition from data-supported interpolation to unconstrained extrapolation, in which predictive uncertainty was no longer guided by experimental evidence, and physically consistent trends could not be guaranteed. Accordingly, while the model may produce smooth numerical outputs beyond the training bounds, its use for quantitative evaluation or design-oriented decision making outside the validated domain was not recommended without additional experimental data and subsequent model retraining. Although the present framework was developed for MWCNT-NFs dispersed in a H₂O–EG (50:50) base fluid, the overall methodology can be extended to other NF systems provided that sufficient experimental data are available for retraining and validation. Nevertheless, changes in NP morphology, base-fluid composition, particle–fluid interactions, and thermophysical nonlinearities may alter the learned input–output relationships and thus require recalibration of the predictive model on a system-specific basis.

### Levenberg-Marquardt (LM)

The ANN was trained using the LM algorithm, which is widely recognized for its efficiency and robustness in nonlinear regression problems with small datasets and low-dimensional input spaces. In the present study, the relationship between thermophysical properties and the governing variables was defined over a compact domain, making LM particularly suitable due to its rapid convergence and numerical stability^32,33^. The LM algorithm operated as a hybrid optimization scheme that integrated the global stability of gradient descent with the fast local convergence of the Newton–Raphson method^32^. During training, the network weights and biases were iteratively updated to minimize the sum-of-squares error between the predicted and experimental outputs. Rather than explicitly computing the Hessian matrix, LM approximated second-order curvature information using the Jacobian of the error function, thereby significantly reducing computational complexity while preserving essential details on the error surface^32^. Compared with first-order training algorithms, such as conventional gradient descent, scaled conjugate gradient, or resilient backpropagation, the LM algorithm generally achieved faster convergence and improved accuracy for compact feedforward neural networks with a limited number of parameters. Moreover, the adaptive adjustment of the damping parameter in LM provided effective control over the step size, enhancing convergence stability and reducing sensitivity to local minima or noise in the training data^34,35^. Given the modest size of the experimental dataset and the relatively simple ANN architecture employed, the use of more computationally intensive or heavily regularized training algorithms was not warranted. Instead, LM offers an optimal balance between computational efficiency and predictive accuracy and has been extensively adopted in thermophysical property modeling and related engineering applications.

1 $$f=\sum\limits_{{i=1}}^{m} {e_{i}^{2}}$$

In Eq. (1), m is the number of training samples, while the Jacobian captures the sensitivity of output errors to parameter variations.

2 $${J_{i,j}}=\frac{{\partial {e_i}}}{{\partial {W_i}}}$$

Equation (4) provides the gradient vector for the descent direction.

3 $$\nabla f=2{J^T}.e$$

Equation (5) derives an approximate Hessian from the error vector, enabling curvature-based refinement of parameters.

4 $$H \approx 2{J^T}.J.\lambda I$$

In Eq. (4), the damping coefficient λ regulates the approximation to keep the Hessian positive definite and to prevent instability. Larger λ values yield updates similar to those of gradient descent, whereas smaller values accelerate convergence by leveraging curvature information. This adaptive mechanism allows LM to maintain efficiency, accuracy, and stability in neural network training, even when data are limited or noisy. The ANN training process used the LM algorithm’s default adaptive parameter update strategy, with the damping coefficient automatically adjusted during optimization to ensure stable convergence and prevent divergence.

5 $${W^{(i+1)}}={W^{(i)}} - {({J^{(i)T}}.{J^{(i)}}.{\lambda ^{(i)}}I)^{ - 1}}.(2{J^{(i)T}}.{e^{(i)}})$$

**Fig. 4:**
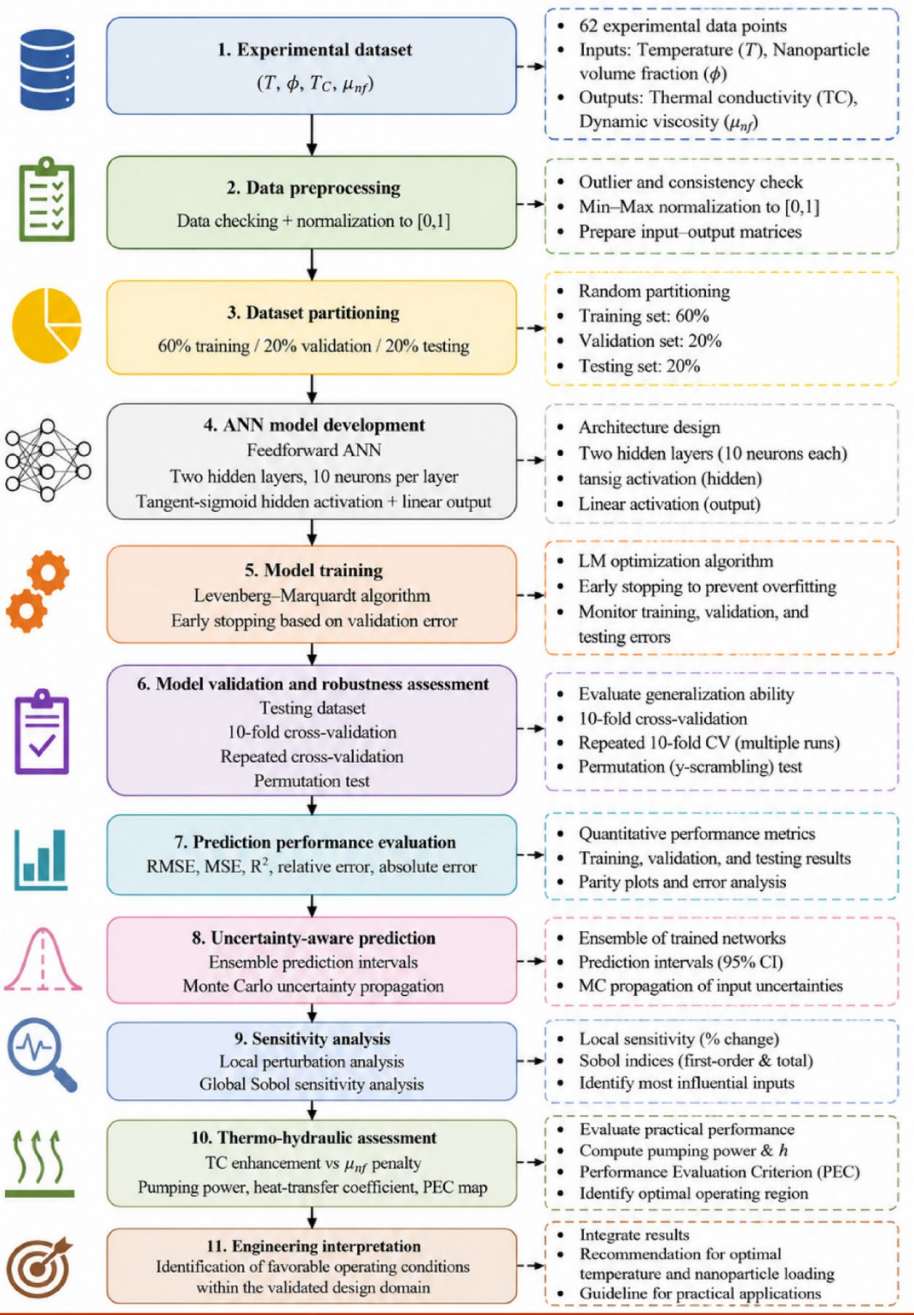
Workflow of the proposed ANN-based predictive framework, including dataset preparation, ANN training, robustness assessment, uncertainty quantification, sensitivity analysis, and thermo-hydraulic evaluation.

The overall workflow of the proposed ANN-based predictive framework is schematically illustrated in Fig. 4.

## Performance indicator

In evaluating the performance of ANNs, the process usually begins by establishing a systematic framework to compare the model’s predictions with the corresponding values in a benchmark dataset. Such a comparison forms the foundation for empirical validation, enabling assessment of the model’s capacity to reproduce the statistical dependencies and hidden nonlinearities present in the data. For datasets characterized by non-stationarity or high structural complexity, selecting appropriate evaluation metrics became particularly important, as these metrics directly determined the reliability of the convergence assessment and the network’s generalization potential. To achieve this, various statistical indices were commonly applied, each emphasizing a different dimension of predictive error. Among them, Mean Squared Error (MSE) and the correlation coefficient (R) were most frequently utilized, providing complementary insights into error variance, prediction deviation magnitudes, and the degree of agreement between predicted and observed outcomes. Moreover, analyzing these indicators separately across training, validation, and testing phases was essential for identifying issues, such as overfitting or underfitting, thereby ensuring a balanced evaluation of model performance.

### The MSE

In regression-oriented machine learning, MSE was widely regarded as a fundamental performance metric, particularly for evaluating neural network models. An aggregate indicator, the MSE was the average of the squared differences between predicted and actual values, reflecting the model’s overall accuracy. Due to the squaring operation applied to each residual, this metric inherently magnified the contribution of large errors, making it highly sensitive to significant deviations. From an operational perspective, the process involved computing the error for each data point as the difference between the model output and the ground truth. These residuals were then squared, a transformation that both removed the directional sign of deviation and increased the relative weight of larger discrepancies^36^. The resulting squared errors were accumulated and subsequently normalized by dividing by the total number of instances in the evaluation dataset, producing a single scalar index. This value served as a robust and interpretable measure of predictive fidelity, and was formally expressed as^37^:

6 $$MSE=\frac{{\sum\limits_{{i=1}}^{n} {{{({P_{oi}} - {P_{si}})}^2}} }}{n}$$

### The R

The R serves as a fundamental statistical index for measuring both the strength and direction of the linear association between the predicted outputs of a regression model and the actual reference values. By capturing not only the magnitude of the relationship, but also its polarity, R effectively characterizes the extent to which the model preserves the inherent linear structure of the target domain^38^. The calculation begins with mean-centering of both the predicted and observed datasets, after which their covariance is computed and normalized by the product of their standard deviations. This yields a unit-free measure constrained to the interval (-1,1). An R value of + 1 indicates perfect positive correlation, where variations in the predictions precisely mirror those of the actual values in both magnitude and direction. Conversely, *R* = -1 signifies a perfect negative relationship, with increases in the predicted values corresponding exactly to decreases in the ground truth. Coefficients near zero indicate negligible linear association, suggesting that the model outputs fail to capture the directional patterns in the reference data and thus do not adequately encode the underlying linear dependencies.

7 $$R=\frac{{\sum\limits_{{i=1}}^{n} {({P_{si}} - \overline {{{P_s}}} )({P_{oi}} - \overline {{{P_o})}} } }}{{\sqrt {\sum\limits_{{i=1}}^{n} {{{({P_{si}} - \overline {{{P_s}}} )}^2}\sum\limits_{{i=1}}^{n} {({P_{oi}} - {{\overline {{{P_o})}} }^2}} } } }}$$

## K-fold cross-validation

**Fig. 5:**
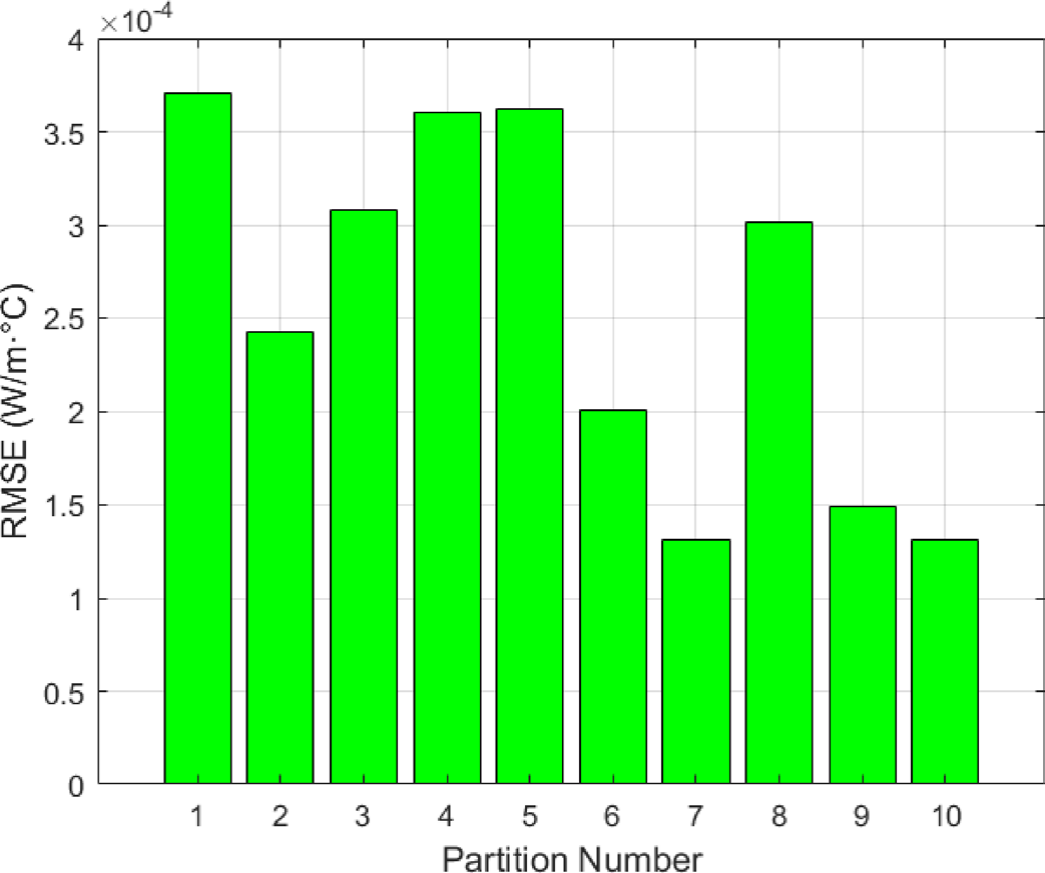
RMSE of TC test data corresponding to 10 partitions created by K-fold Cross Validation.

To ensure a rigorous and unbiased evaluation of the developed predictive framework, k-fold cross-validation was employed. In this method, the dataset was randomly partitioned into k mutually exclusive, equally sized subsets (folds). For each iteration, the model was trained on k − 1 folds and validated on the remaining fold. This procedure was repeated until each fold was used once for validation, and the final performance indices were averaged across all iterations. Such a strategy effectively minimized variance across a single train–test split, reduced the risk of overfitting, and provided a reliable measure of the model’s generalization capability. In this study, a 10-fold cross-validation scheme was used to assess the neural network’s accuracy in predicting TC for HNFs. RMSE values for TC ranged from 1.31 × 10^− 4^ W/m·°C to 3.71 × 10^− 4^ W/m·°C, with an overall mean of 2.56 × 10^− 4^ W/m·°C across the folds. These findings demonstrated that the model can capture the nonlinear dependencies governing TC with high precision. The distribution of RMSE values across all partitions, which showed the consistency and robustness of the predictive framework, is shown in Fig. 5.

**Fig. 6:**
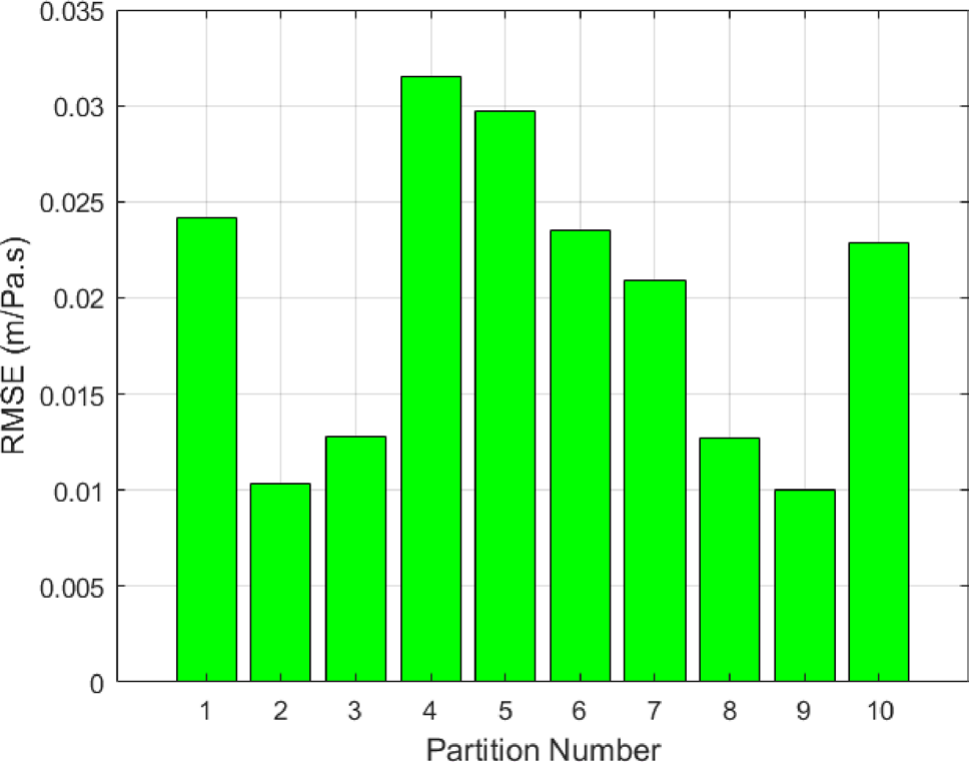
RMSE of µ_nf_ test data corresponding to 10 partitions created by K-fold cross validation.

A similar cross-validation analysis was conducted for µ_nf_ model, and the results are presented in Fig. 6. The 10-fold procedure revealed that RMSE values for µ_nf_ prediction ranged from 0.010 mPa·s to 0.031 mPa·s, with an average of 0.0198 mPa·s across all folds. Compared with TC, the RMSE values of µ_nf_ were relatively larger, which can be attributed to µ_nf_’s greater sensitivity to changes in NP-φ and T. Nonetheless, the overall low magnitude of errors demonstrated that the ANN-based model reproduced the experimental µ_nf_ trends with sufficient reliability. The variability in RMSE across folds further underscored the importance of cross-validation in capturing dataset heterogeneity and ensuring that the predictive framework remained stable across different statistical partitions. These findings showed the robustness of the proposed ANN architecture in modeling both transport properties of HNFs.

**Fig. 7:**
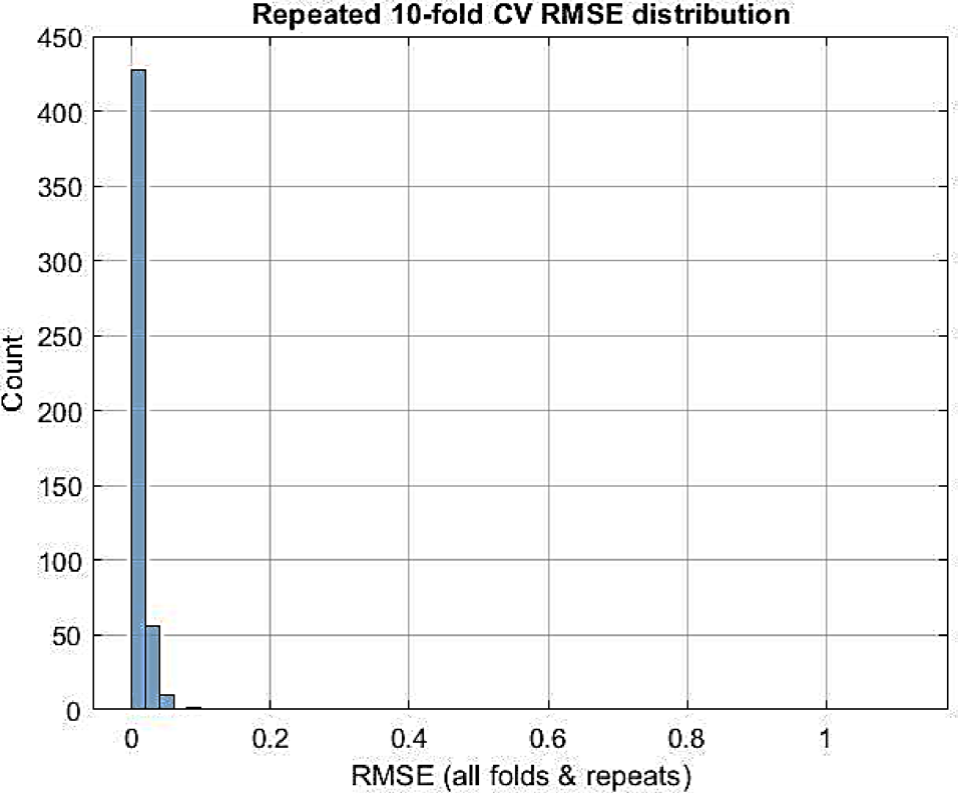
RMSE distribution obtained from repeated 10-fold cross-validation of the ANN model.

While the single-run 10-fold cross-validation results presented above demonstrated the ANN’s high predictive accuracy for both TC and µ_nf_, the limited dataset size necessitated a more stringent assessment of model robustness and potential optimism in error estimation. In particular, the inherent variability of small folds may yield favorable partitions that underestimate the true generalization error. To further examine the stability of reported performance and to ensure that the low RMSE values were not artifacts of a specific data split or chance correlations, the validation strategy was extended to include repeated cross-validation and permutation-based testing. Figure 7 illustrates the distribution of RMSE values obtained from the repeated 10-fold cross-validation procedure applied to the ANN model. By repeating 10-fold partitioning 50 times, a total of 500 independent test evaluations were generated, each based on folds containing three samples. The resulting RMSE distribution showed a strong concentration at low error values, indicating that the ANN’s predictive accuracy was consistently preserved across repeated resampling of the dataset. Quantitatively, the model achieved a mean RMSE of 0.0177 and a standard deviation of 0.0516, with a 95% bootstrap confidence interval for the mean RMSE of [0.0146, 0.0230]. The relatively broad spread of individual RMSE values indicated fold-to-fold variability inherent to small-sample validation rather than systematic instability in the ANN. Importantly, the narrow confidence interval of the mean confirmed that the expected generalization error remains low and statistically stable, reinforcing the robustness of the proposed predictive framework beyond a single cross-validation run.

**Fig. 8:**
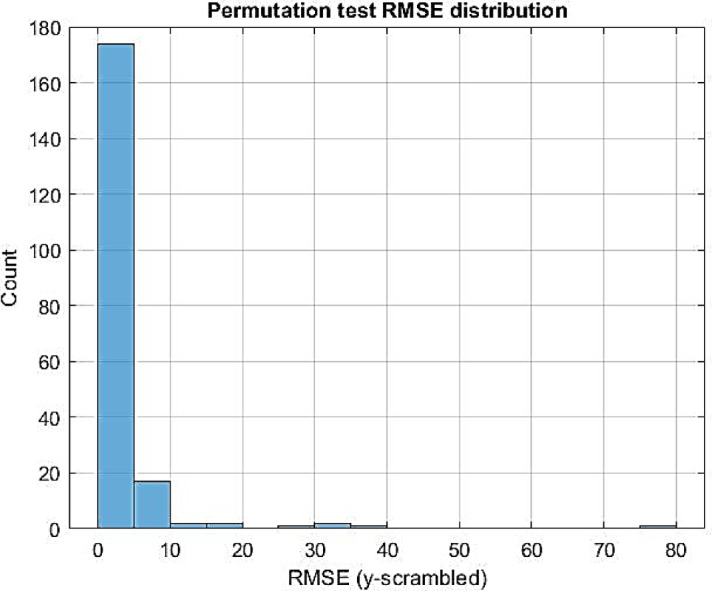
RMSE distribution obtained from the permutation (y-scrambling) test.

Figure 8 presents the RMSE distribution obtained from the permutation (y-scrambling) test, which served as a stringent control to detect optimistic bias and spurious correlations. In this analysis, the target values were randomly permuted while preserving the input structure and the original repeated 10-fold cross-validation protocol. In stark contrast to the original model, the permutation test resulted in substantially larger prediction errors, with a mean RMSE of 3.57 and a standard deviation of 7.16, and a broad 5th–95th percentile range of [0.815, 8.86]. Furthermore, the empirical probability of achieving an RMSE equal to or lower than that of the original ANN under permutation was effectively zero (p ≈ < 0.0000). This pronounced separation between the RMSE distributions of original and scrambled targets provided compelling statistical evidence that the ANN performance was not driven by random structure or data leakage. Instead, it confirmed that the low prediction errors arose from a meaningful and physically consistent relationship between the input variables (T and NP-φ) and the predicted thermophysical properties.

### Benchmark comparison with alternative regression models

To further evaluate whether the adopted ANN architecture provided meaningful predictive advantages relative to simpler regression approaches, additional benchmark comparisons were conducted using linear regression, second-order polynomial regression, Gaussian process regression (GPR), and support vector regression (SVR). All models were trained and evaluated on identical normalized datasets and using 10-fold cross-validation to ensure a statistically consistent comparison. The resulting performance metrics are summarized in Table 1. The benchmark analysis revealed that the ANN framework achieved the highest predictive accuracy for TC estimation, yielding the lowest RMSE and MAE values, along with the highest R, among all examined models. This behavior indicated that the ANN architecture captured the coupled nonlinear dependence of TC on T and NP-φ more effectively, whereas simpler regression models exhibited reduced flexibility in representing the complex thermophysical interactions governing heat transport in MWCNT-NFs.For µnf prediction, polynomial regression and GPR yielded slightly lower RMSE values than the ANN framework. Nevertheless, the ANN model maintained high predictive accuracy and stable generalization, with R² remaining above 0.996. This outcome suggested that, although compact nonlinear regressors may adequately approximate isolated µ_*nf*_ behavior within limited operating domains, the ANN framework provided a more comprehensive nonlinear structure capable of supporting uncertainty quantification, sensitivity analysis, and coupled thermo-hydraulic interpretation within a unified predictive methodology.

**Table 1:** Benchmark comparison of regression and machine-learning models for prediction of TC and µnf under identical 10-fold cross-validation conditions.

Benchmark model	TC (RMSE)	TC (MAE)	TC (R)	*µ*_*nf*_ (RMSE)	*µ*_nf_ (MAE)	*µ*_nf_ (R)
Linear Regression	0.00065	0.00051	0.994	0.0821	0.0596	0.981
Polynomial Regression	0.00034	0.00024	0.998	0.0159	0.0132	0.999
GPR	0.00055	0.00046	0.996	0.0148	0.0121	0.999
SVR	0.0011	0.00083	0.983	0.0772	0.0531	0.983
ANN	0.00022	0.00019	0.999	0.0348	0.0224	0.996

## Prediction results and analysis

### Thermal–hydraulic performance of a representative microchannel heat sink

The thermal–hydraulic performance of working fluid in a microchannel heat sink was governed by the concurrent variation of TC and µ_nf_ with operating T and φ. Accordingly, the present analysis used a discrete set of operating points (T, ϕ), spanning a T interval of approximately 30–80 °C and a dilute-to-moderate range of φ^31^. For each operating point, the effective TC (T, ϕ) and the µ_nf_ (T,ϕ) were specified for both the base fluid and the NF. This paired dataset enabled a consistent, point-wise evaluation of HT enhancement and hydraulic penalty under identical operating conditions. To convert (k, µ) into practical design implications for microchannel cooling, these thermophysical inputs were coupled with standard internal-flow relations for laminar transport in rectangular ducts. Specifically, for a representative microchannel of width W, height H, and length L, the hydraulic diameter is defined as:

8 $${D_h}=\frac{{2WH}}{{W+H}}$$

The mean flow velocity is obtained from the Reynolds number definition,

9 $$V=\frac{{\mu \operatorname{Re} }}{{D{\rho _h}}}$$

In microchannel heat sinks operating under laminar flow conditions, µ_nf_ effects dominated the hydraulic resistance. Although NP addition may enhance TC, the associated increase in dynamic µ_nf_ results in higher frictional losses, manifested as increased pressure drop and pumping power at a fixed Reynolds number. This penalty was particularly pronounced at lower Ts, where the base-fluid µ_nf_ was intrinsically high, and the suspension-induced thickening further amplified viscous dissipation. For fully developed laminar flow in a rectangular duct, the Darcy friction factor was evaluated from the Poiseuille number formulation. The resulting pressure drop along the microchannel is expressed as

10 $$\Delta P=f\frac{L}{{{D_h}}}\frac{{\rho {V^2}}}{2}$$

and the required pumping power is obtained from:

11 $${P_{pump}}=\Delta P\dot {V}$$

**Fig. 9:**
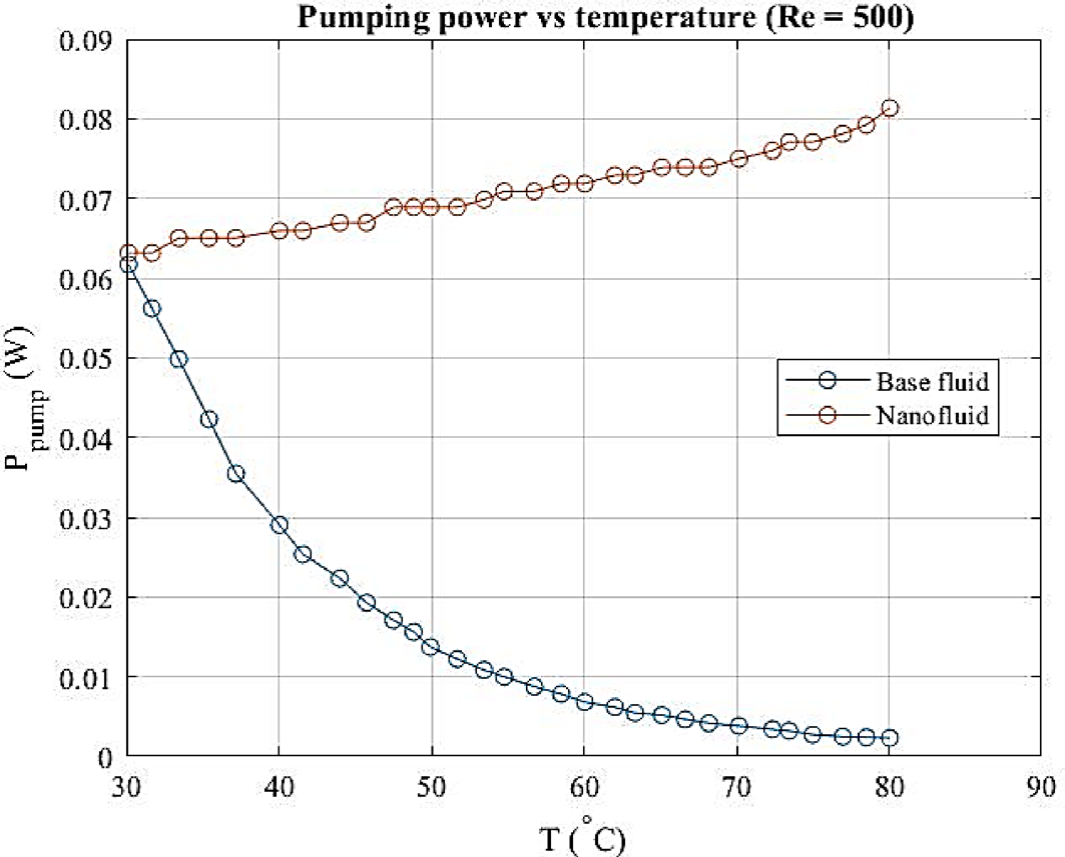
Pumping power as a function of T for the base fluid and NF at Re = 500.

**Fig. 10:**
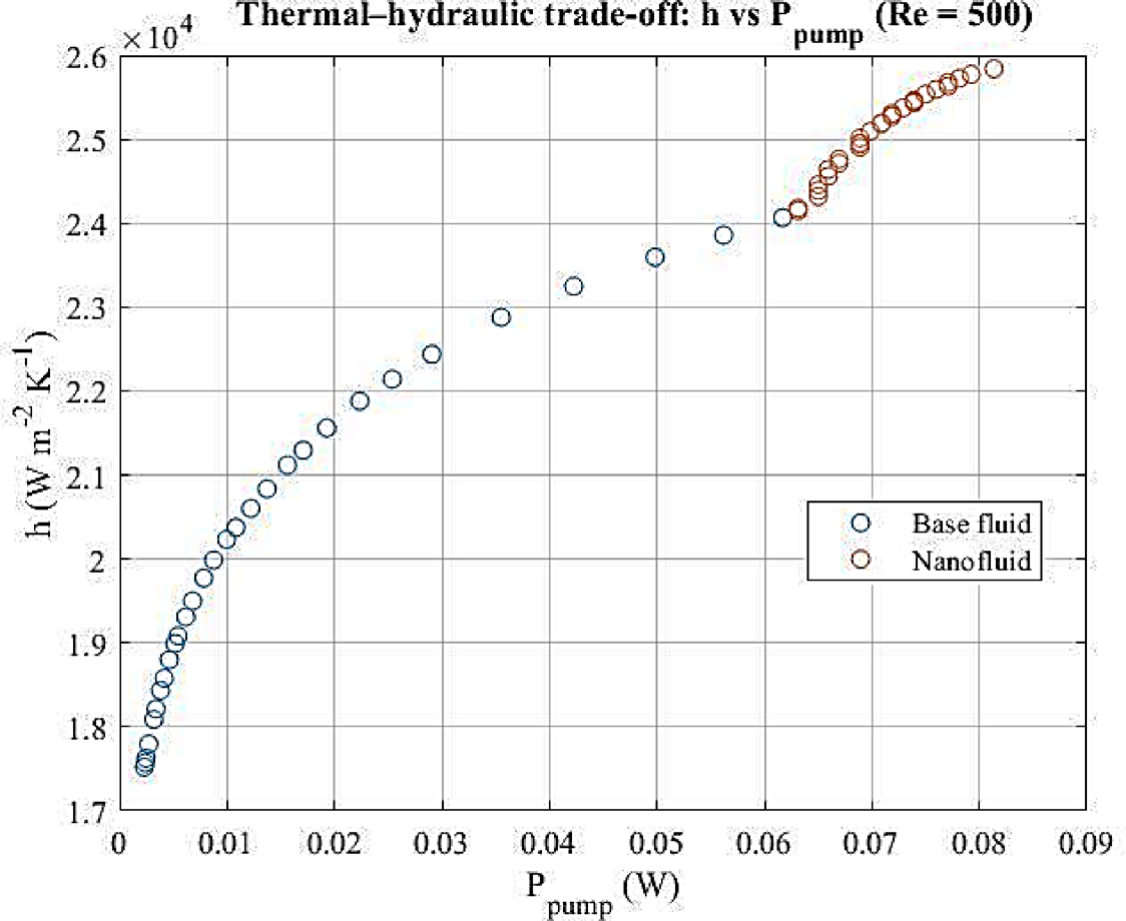
Thermal–hydraulic trade-off between HT coefficient and pumping power at Re = 500.

**Fig. 11:**
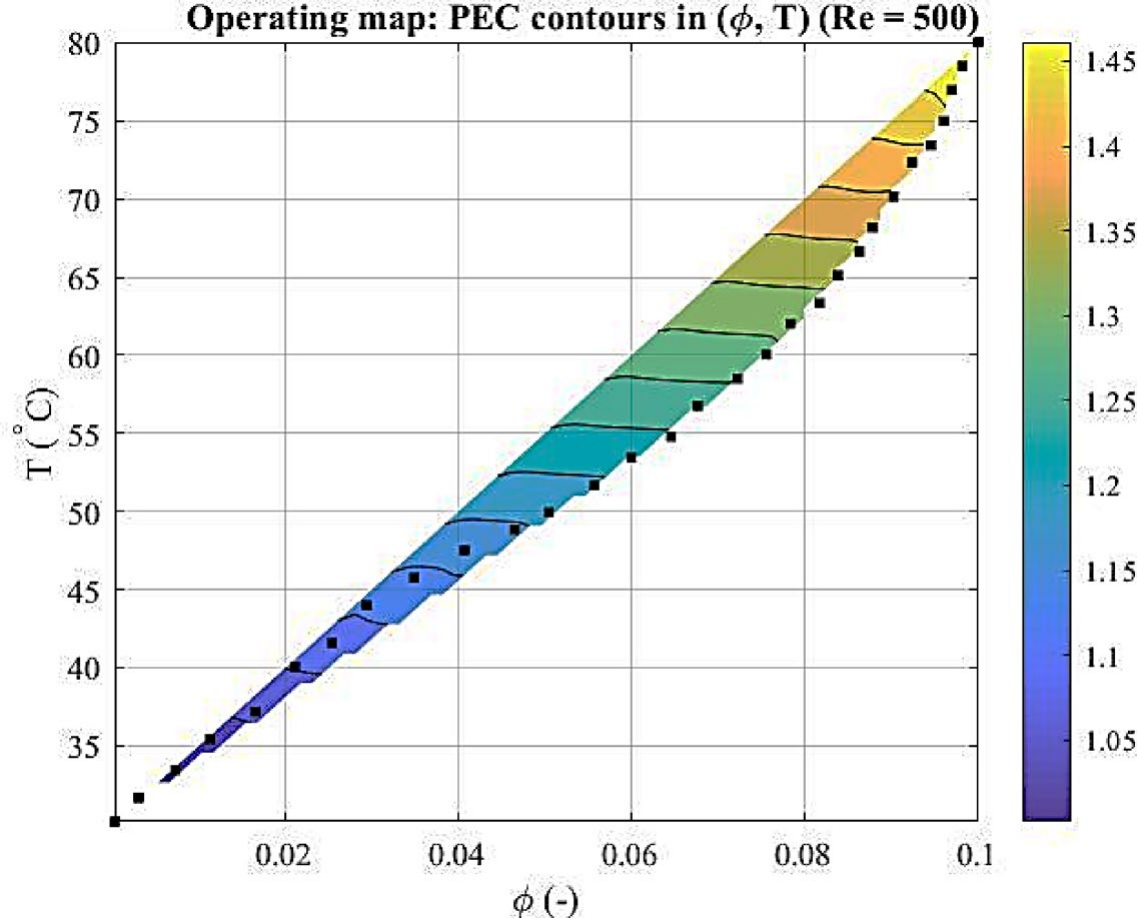
Operating map in the (ϕ, T) plane based on PEC contours at Re = 500.

Where V˙denotes the volumetric flow rate. These relations explicitly demonstrate how variations in µ (T, ϕ) translate into energetic penalties, emphasizing that TC enhancement alone is insufficient for evaluating cooling performance in confined flows (Figs. 9, 10, 11).

The convective HT performance was quantified using the HT coefficient h, which was derived from the Nusselt number and the effective TC. For laminar flow in rectangular microchannels, the Nusselt number was evaluated from established correlations consistent with the imposed thermal boundary condition. The HT coefficient is then obtained as

12 $$h=\frac{{Nu.k}}{{{D_h}}}$$

Due to the monotonic increase of TC with T and φ, the HT coefficient showed a corresponding upward trend. However, this enhancement must be interpreted in conjunction with the hydraulic penalties discussed previously, since increased µ_nf_ can offset, or even outweigh, the thermal gains when assessed at the system level (See Fig. 10).

To provide an actionable design perspective, thermal enhancement and hydraulic penalty were evaluated simultaneously using thermal–hydraulic trade-off charts. For each representative Reynolds number, the relationship between the HT coefficient and pressure drop was illustrated by plotting h as a function of ΔP. This representation directly showed whether improvements in convective performance were achieved at an acceptable hydraulic cost. In parallel, the relationship between h and pumping power provides an energy-aware metric that was particularly relevant for forced-convection microchannel cooling systems. To consolidate these competing effects into a single performance indicator, a performance evaluation criterion (PEC) is defined as

13 $$PEC=\frac{{(Nu/N{u_0})}}{{{{(f/{f_0})}^{\frac{1}{3}}}}}$$

In Eq. (13), the subscript 0 denotes the corresponding base-fluid values evaluated at the same Reynolds number and T. Values of PEC > 1 indicate that the HT enhancement outweighs the frictional penalty in a net sense. The recommended operating region was therefore identified as the domain in the (T, φ) space where PEC exceeded unity while avoiding excessive pressure drop or pumping-power requirements. From an engineering perspective, the proposed predictive framework can support preliminary thermo-hydraulic design and operating-condition assessment in compact thermal management systems. By simultaneously evaluating conductivity enhancement and µ_*nf*_-induced hydraulic penalties, the model enables rapid identification of operating regions that offer favorable heat-transfer performance without excessive pumping power. Such capability is particularly relevant for microchannel cooling devices, compact heat exchangers, and energy-efficient thermal systems, where small variations in fluid properties can significantly influence overall system performance, flow resistance, and operational energy consumption (See Fig. 11).

### Comparison of ANN vs. classical correlations

To quantitatively benchmark the predictive performance of the proposed ANN framework, a head-to-head comparison was conducted against representative classical correlations for TC and µ_nf_, namely the Maxwell-type and Brinkman-type models. These correlations, rooted in effective medium theory and dilute suspension assumptions, were widely used as reference baselines for NF property estimation. All models were evaluated under identical repeated 10-fold cross-validation partitions, ensuring that performance differences arose solely from modeling capability rather than from data partitioning or parameter leakage.

#### TC comparison

The comparative results are reported in Table 2 demonstrate that the ANN model provided substantially more accurate and stable TC predictions than the Maxwell-type correlation. The classical formulation exhibited pronounced deviations from experimental trends and substantial variability across validation folds, suggesting limited robustness when applied to MWCNT-based NFs. This behavior showed the restrictive assumptions underlying the Maxwell model, particularly its inability to account for non-spherical particle morphology, T-dependent effects, and interaction-induced nonlinearities. In contrast, the ANN consistently captured the coupled influence of T and φ, resulting in markedly improved, statistically stable predictive performance.

**Table 2:** Head-to-head comparison between ANN and Maxwell-type correlation for TC prediction under identical repeated 10-fold cross-validation.

Model	RMSE_mean_	RMSE_Std_	MAE_Mean_	MAE_Std_	*R*_Mean_	*R*_Std_
ANN	0.0002469	0.0001074	0.0002131	9.26 × 10⁻⁵	0.99798	0.00445
Maxwell	0.085329	0.011825	0.078655	0.014028	– 211.75	309.08

#### µ_nf_ comparison

A similar trend was observed for µ_nf_ prediction, as summarized in Table 3. Brinkman-type correlation showed limited agreement with experimental data and substantial dispersion in prediction accuracy, underscoring its inadequacy for describing the complex rheological behavior of CNT-based NFs. ANN framework, however, demonstrated robust generalization across repeated resampling, highlighting its ability to represent nonlinear µ_nf_ variations arising from combined thermal and concentration effects. These findings confirmed that data-driven modeling provided a more reliable description of µ_nf_ behavior than classical analytical correlations under the investigated conditions.

**Table 3:** Head-to-head comparison between ANN and Brinkman-type correlation for μ_nf_ prediction under identical repeated 10-fold cross-validation

Model	RMSE_mean_	RMSE_Std_	MAE_Mean_	MAE_Std_	*R*_Mean_	*R*_Std_
ANN	0.018001	0.07217	0.015109	0.05022	0.96873	0.61862
Brinkman	0.81762	0.17801	0.73623	0.194	– 3.2086	4.9207

#### Overall assessment

**Fig. 12:**
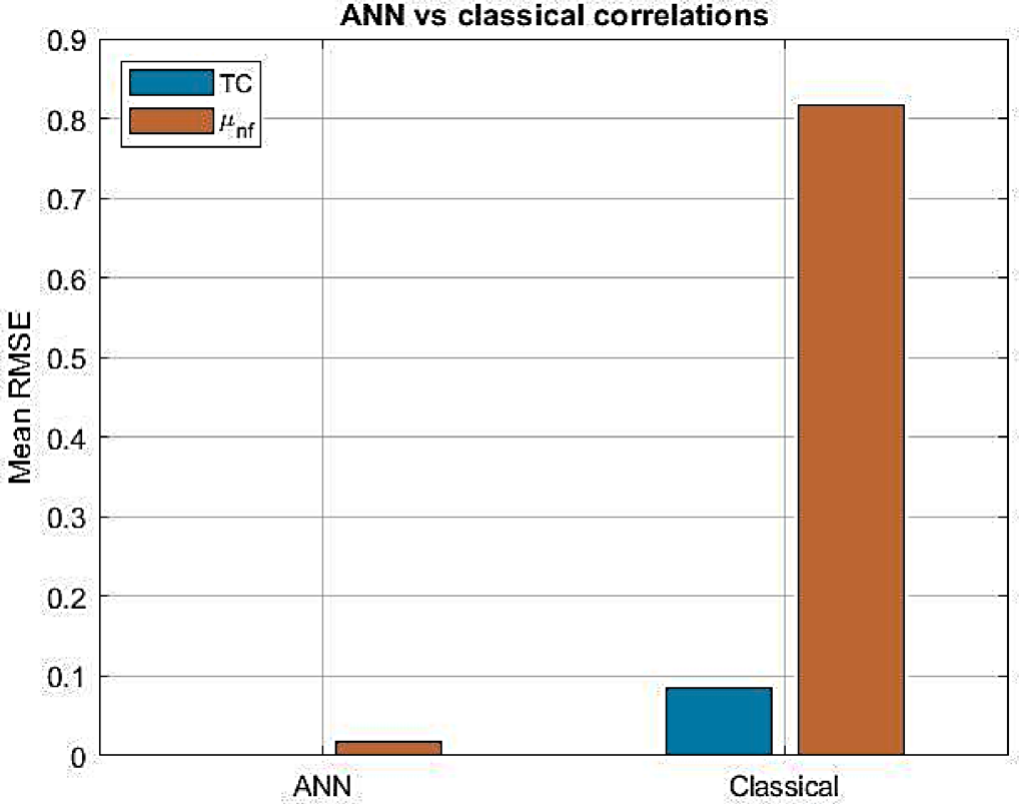
Comparison of mean RMSE values obtained from ANN and classical correlations (Maxwell-type for TC and Brinkman-type for µ_nf_) across repeated 10-fold cross-validation.

The aggregated comparison presented in Fig. 12 clearly highlights the systematic superiority of the ANN framework over classical correlations for both thermophysical properties. While Maxwell- and Brinkman-type models remained valuable as simplified, physically interpretable baselines, their predictive capabilities were inherently constrained by idealized assumptions. ANN framework effectively overcame these limitations by learning the underlying nonlinear relationships directly from experimental data, thereby offering a more accurate and robust predictive tool within the validated experimental domain.

### Comparison of different performance functions

**Fig. 13:**
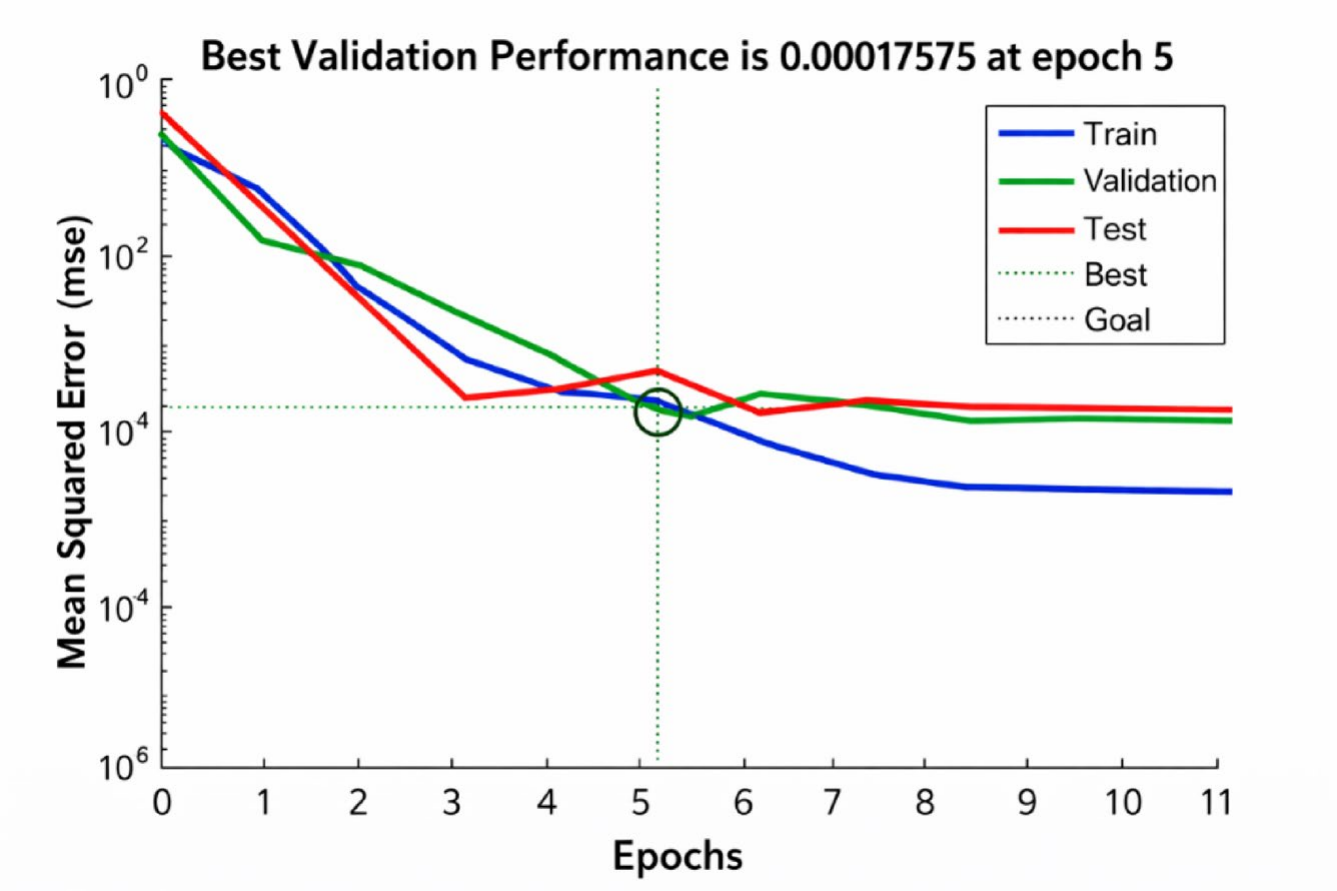
ANN performance diagram during the MSE index optimization stages corresponding to each category of training, validation, and test data of TC.

Figure 13 illustrates the variation in the neural network’s MSE during training to predict TC. A consistent decline in MSE across the training, validation, and testing phases was observed as epochs progressed, reflecting the efficient adjustment of network parameters and its ability to model the nonlinear interactions between the inputs and TC. The best validation performance occurred at epoch 5, where the MSE reached a minimum of 1.7573 × 10⁻⁴ W/m·°C. The corresponding overall, training, and testing MSE values at this stage were 4.0429 × 10⁻⁴ W/m·°C, 3.5971 × 10⁻⁴ W/m·°C, and 7.6654 × 10⁻⁴ W/m·°C, respectively. These results showed that the model’s predictive accuracy was strong in reproducing TC trends across various operating conditions. The close agreement between the training and validation error trajectories confirmed that the network avoided overfitting and retained strong generalization. Moreover, the relatively low testing error indicated that the model can reliably extend its predictions to unseen data points. Collectively, these findings verify the robustness of the adopted ANN architecture for TC estimation and support its suitability as a computational tool for analyzing the coupled effects of T and φ in HNFs.

**Fig. 14:**
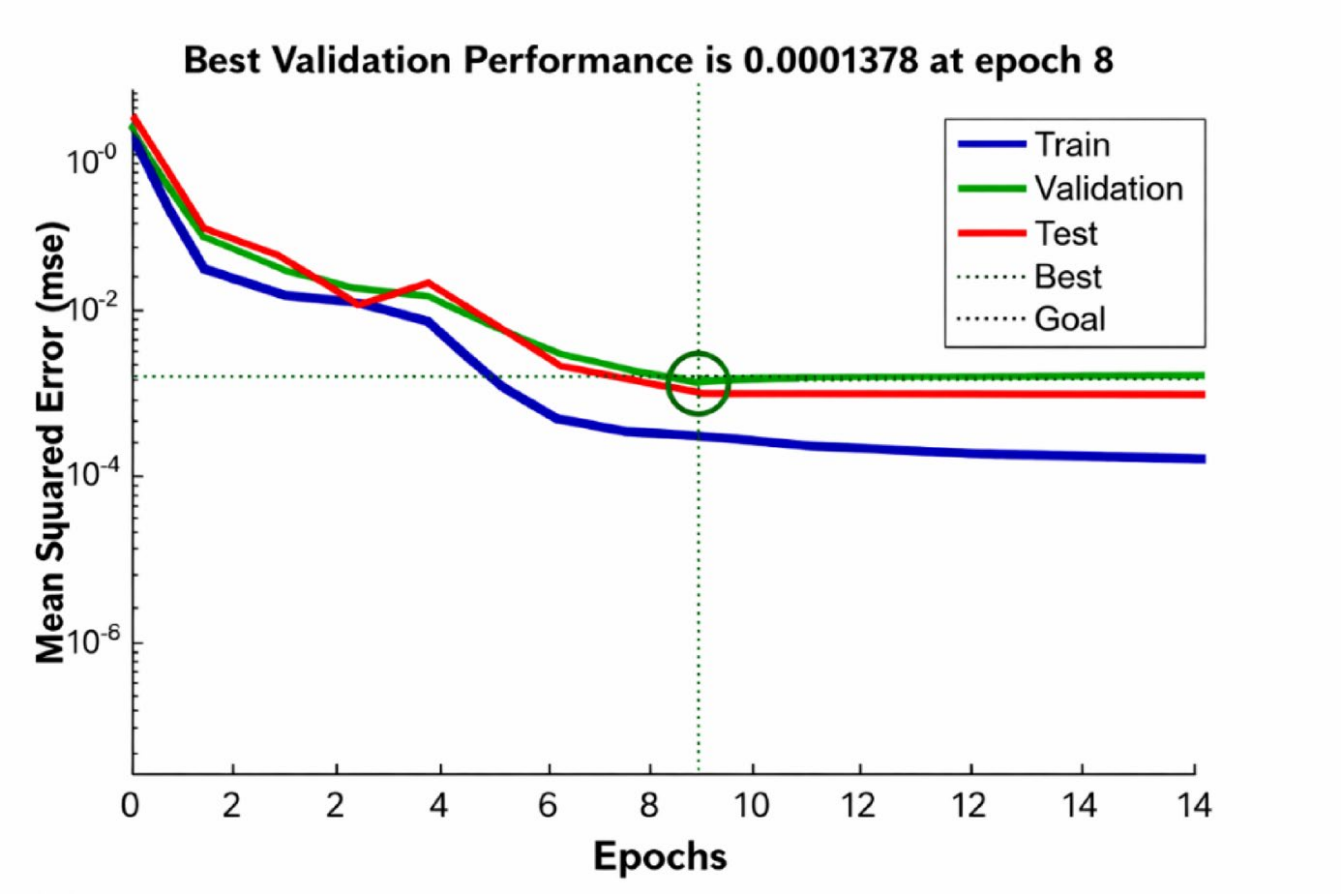
ANN performance diagram during the MSE index optimization stages corresponding to each category of training, validation, and test data of µ_nf_.

Figure 14 presents the MSE evolution during training of the ANN model for µ_nf_. The results demonstrated a consistent reduction in error across the training, validation, and testing phases, reflecting efficient learning and an accurate representation of nonlinear effects. The best validation result was observed at epoch 8 with an MSE of 1.3780 × 10⁻⁴ mPa·s, while the overall, training, and testing errors were 6.5217 × 10⁻⁵ mPa·s, 2.7466 × 10⁻⁵ mPa·s, and 1.0589 × 10⁻⁴ mPa·s, respectively. The close alignment of training and validation curves indicated the absence of overfitting, and the low testing error further confirmed the model’s generalization ability. These outcomes showed the robustness of the ANN framework in predicting µ_nf_ trends of HNFs with high reliability.

**Fig. 15:**
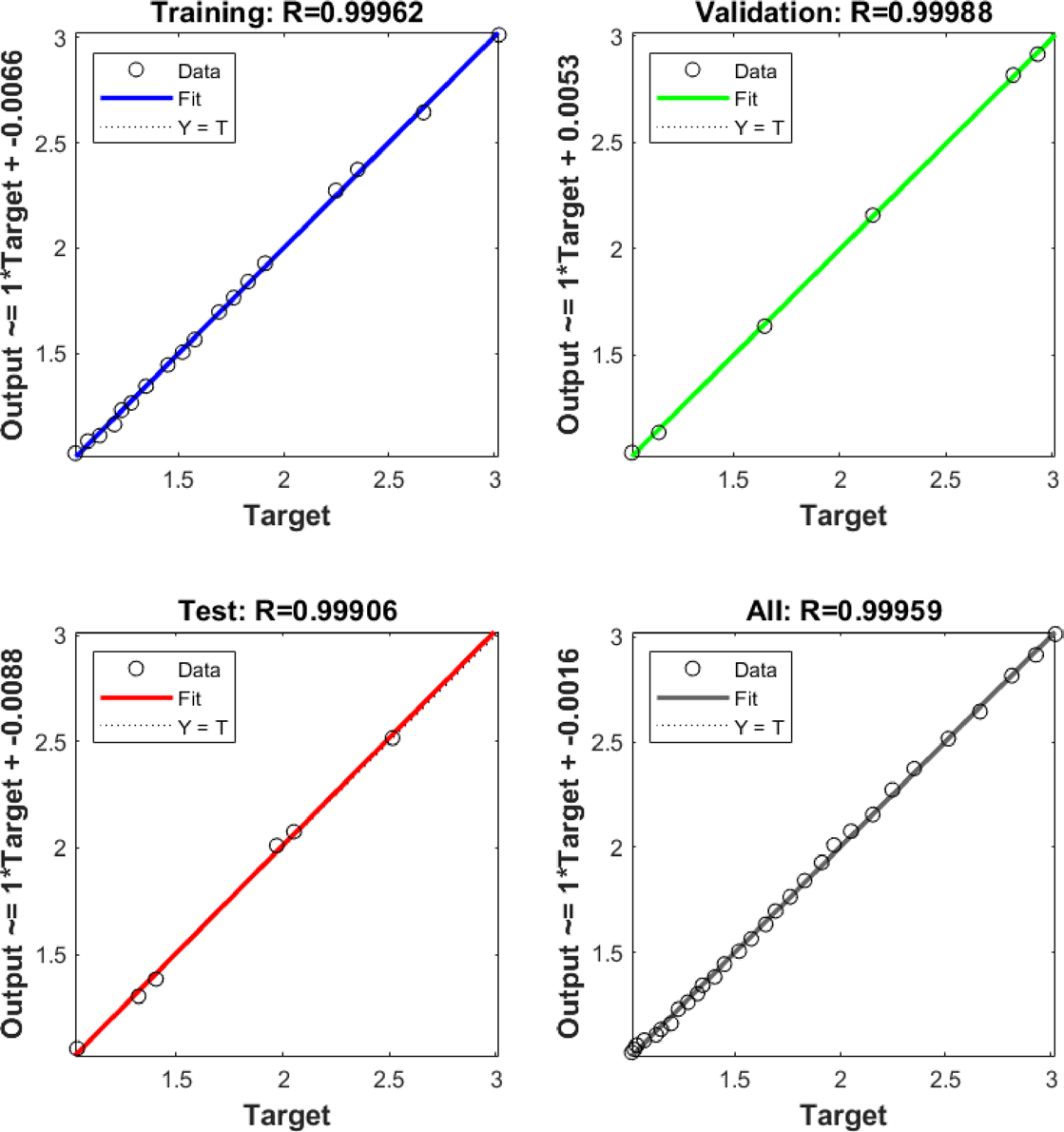
ANN regression diagram corresponding to the TC output for each set of training, validation, and test data.

Figure 15 illustrates the statistical correlation between the predicted and measured TC values obtained from the ANN model. The training phase yielded a *R* = 0.99962, indicating the model’s strong ability to capture the dataset’s intrinsic patterns. In the validation phase, the correlation increased slightly to 0.99988, confirming the framework’s stability when applied to unseen data. For the testing subset, the correlation remained consistently high at 0.99906, providing evidence of reliable extrapolation beyond the training range. Considering all the data together, the overall R was 0.99959, demonstrating that the ANN successfully reconstructed TC’s nonlinear behavior with outstanding precision. Such consistently high correlation values verified that the proposed modeling strategy was both accurate and generalizable.

**Fig. 16:**
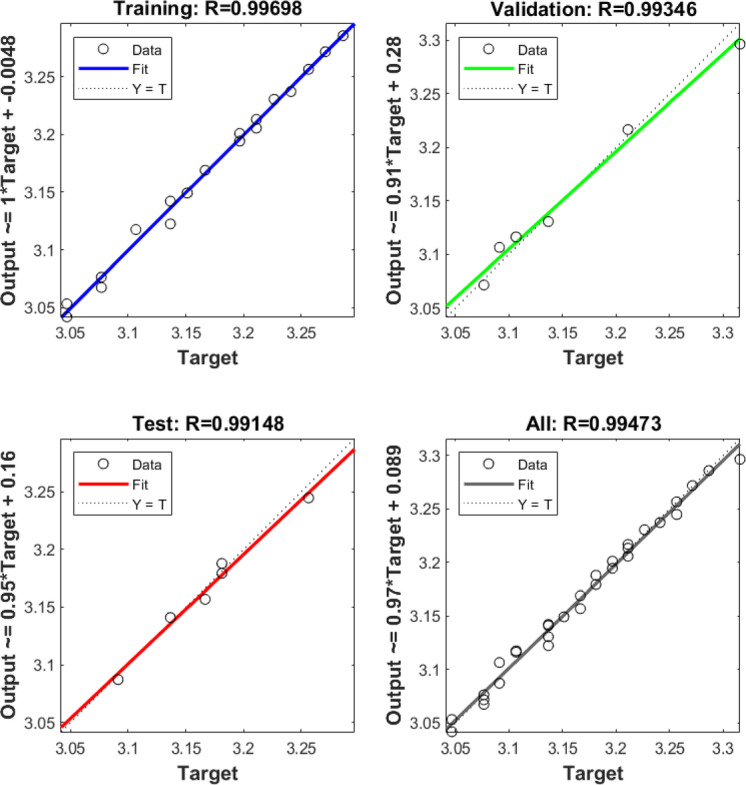
ANN regression diagram corresponding to the µ_nf_ output for each set of training, validation and test data.

As illustrated in Fig. 16, the ANN model’s correlation performance confirmed its ability to reproduce the µ_nf_ behavior with high accuracy. The training dataset achieved an R of 0.99698, indicating that the network effectively captured the nonlinear relationships in the data. The validation subset yielded a correlation of 0.99346, indicating stable generalization to unseen samples. For the testing partition, the R was slightly lower at 0.99148, yet it remained within the range of strong predictive reliability. Across the entire dataset, the R value reached 0.99473, indicating strong agreement between the predicted and experimental values.

**Fig. 17:**
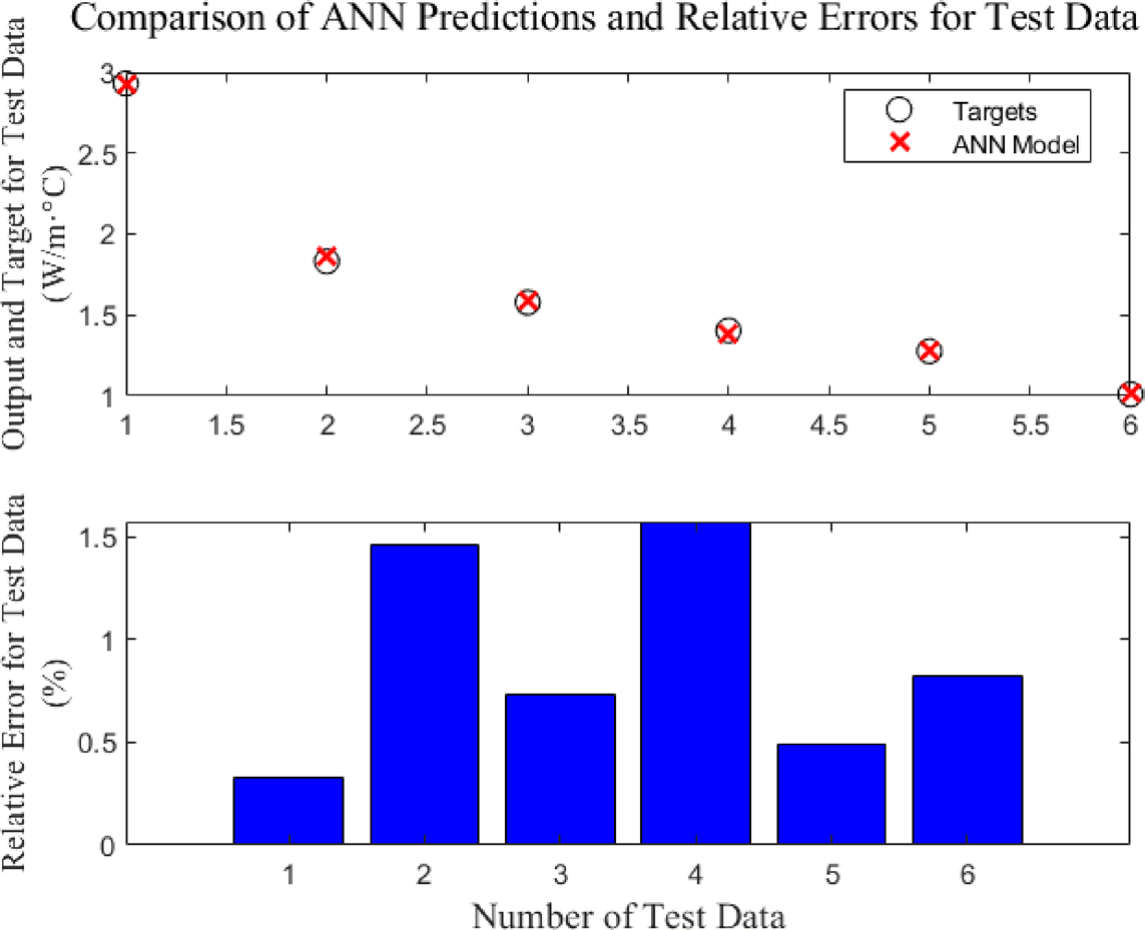
Correspondence graphs and percent error between the actual TC values and the corresponding ANN output, for network test data.

Figure 17 compares the experimental measurements with the ANN-predicted TC values, along with a relative error analysis for the test dataset. The results demonstrated a close correspondence between predicted and observed data, confirming the reliability of the developed model. The relative error fluctuated within a narrow range, from 0.3245% to 1.5737%, indicating that the ANN maintained high precision across all operating conditions. The limited deviation observed not only demonstrated the robustness of the training process but also the model’s ability to capture the nonlinear relationships among φ, T, and TC.

**Fig. 18:**
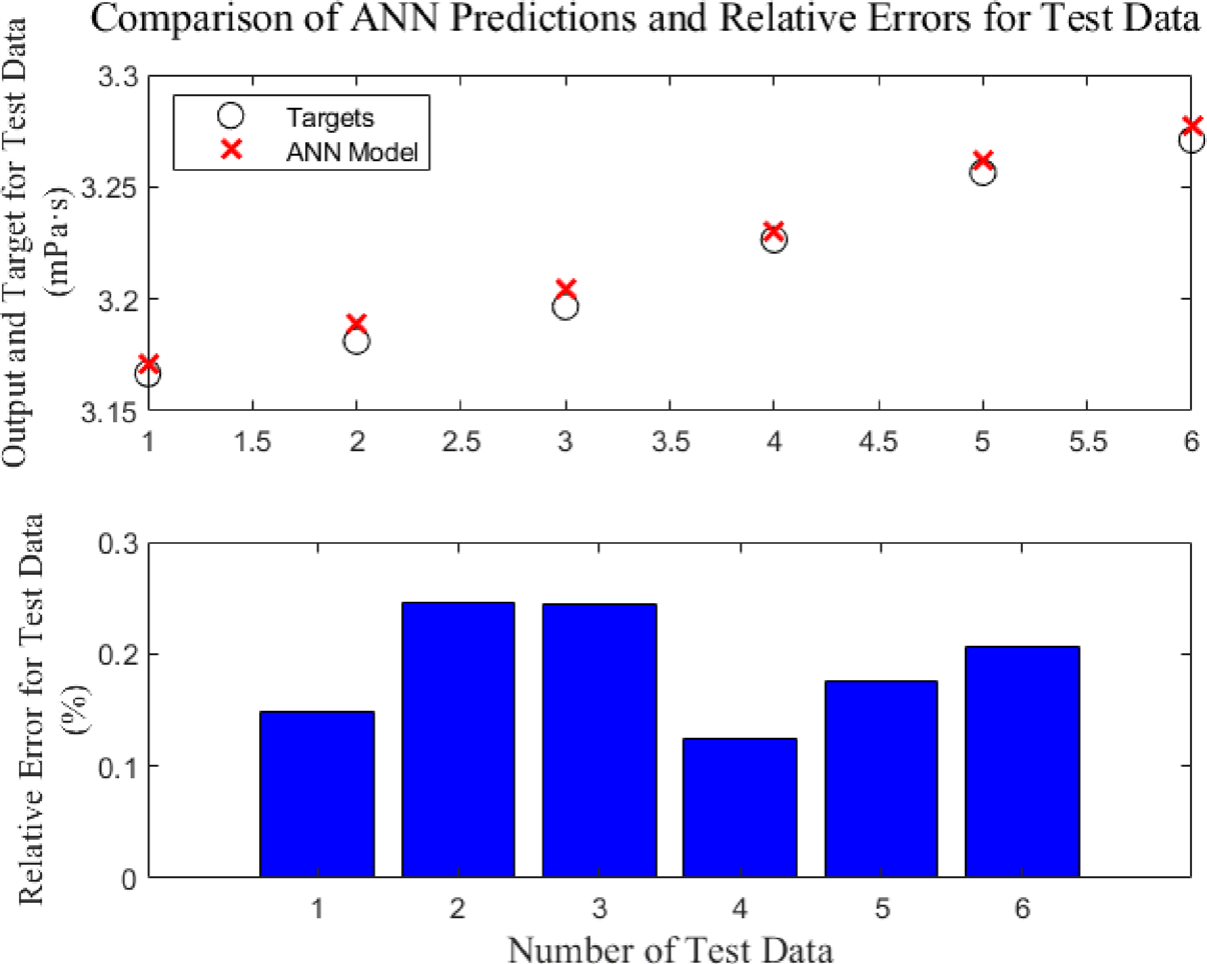
Correspondence graphs and percent error between the actual µ_nf_ values and the corresponding ANN output, for network test data.

Figure 18 illustrates the ANN model’s performance in predicting µ_nf_, with accuracy further assessed via relative error analysis. The comparison between predicted and experimental values showed a very close agreement, confirming the model’s ability to reproduce µ_nf_ behavior under the examined operating conditions. The relative error was consistently low, ranging from 0.1246% to 0.2457%, demonstrating the network’s stability and robustness in capturing the nonlinear rheological characteristics of NF. Such precision underscored the ANN’s suitability for reliable µ_nf_ estimation, providing valuable insights into flow resistance and pumping requirements in practical thermal systems.

**Fig. 19:**
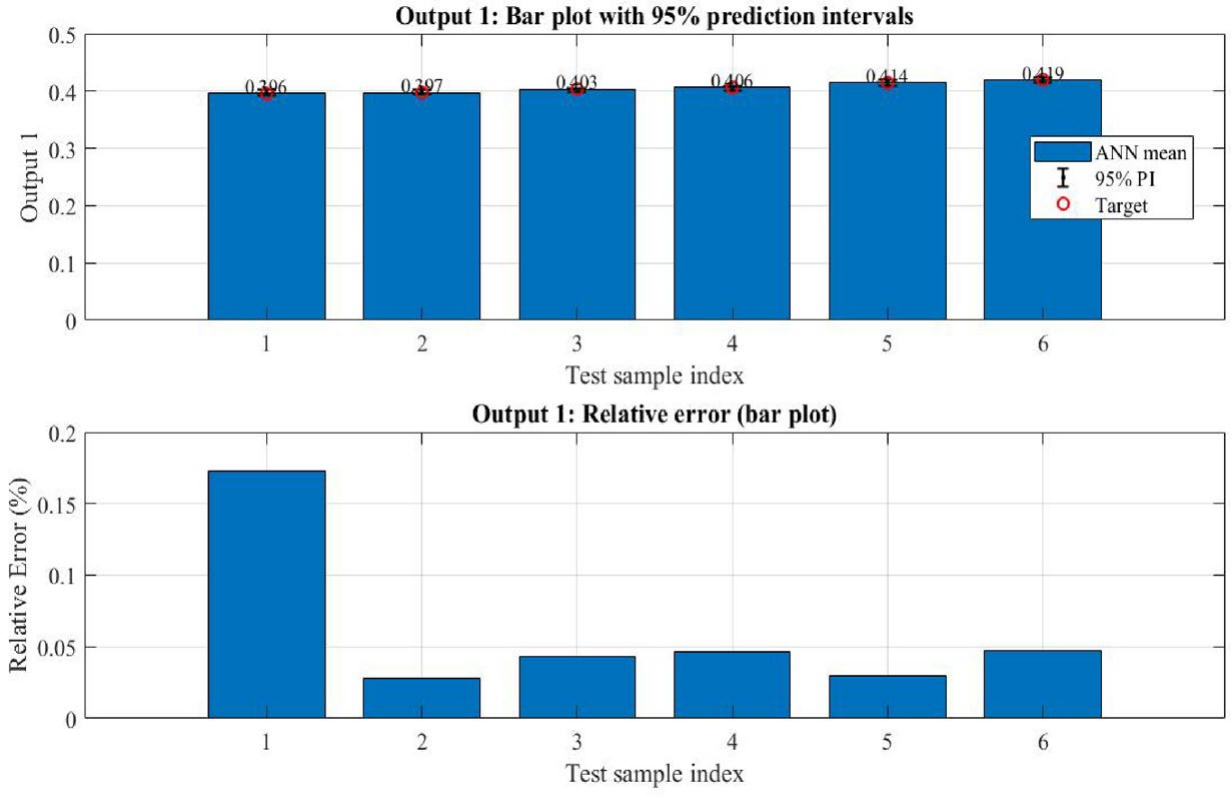
ANN ensemble mean predictions of TC for the test dataset, including 95% prediction intervals obtained via deep ensemble modeling.

The ANN-predicted thermophysical trends remained strongly consistent with the established transport mechanisms governing MWCNT-NFs. The observed enhancement in TC with increasing T and NP-φ can be physically attributed to intensified Brownian motion, improved particle–fluid interfacial energy exchange, and the progressive formation of conductive transport pathways within the H₂O–EG base fluid, generated by the dispersed nanotube network. Increasing T additionally reduced thermal resistance at the particle–fluid interface and facilitated nanoscale energy transport throughout the suspension.In contrast, the increase in µnf primarily originated from enhanced hydrodynamic interactions and momentum-transfer resistance associated with higher nanotube loading. As NP-φ increased, the probability of nanotube–nanotube interactions and localized flow obstructions increased, leading to greater viscous dissipation and stronger resistance to fluid motion. T exerted a partially compensating influence by reducing intermolecular cohesive forces and improving molecular mobility, thereby moderating the µ_*nf*_ increase at elevated operating conditions. The ability of the ANN framework to consistently reproduce these coupled thermo-hydraulic trends demonstrated that the learned input–output relationships remained physically meaningful and aligned with established NF transport mechanisms, rather than representing purely statistical fitting. Figure 19 presents an uncertainty-aware assessment of the ANN model’s performance for TC. The predicted ensemble mean values showed an excellent agreement with the experimental measurements, while the associated 95% prediction intervals, obtained through deep ensemble modeling combined with Monte Carlo propagation of input uncertainties (SD(T) = 1.96% and SD(φ) = 0.91%), remained narrow across the entire test dataset. The confinement of all experimental TC values within the predicted uncertainty bounds indicated both statistical consistency and numerical stability of the proposed model. Moreover, the narrow width of the prediction intervals indicated the relatively low sensitivity of TC to small perturbations in T and NP-φ within the investigated range, confirming that the ANN effectively captured the dominant heat transport mechanisms governing the system (Tables 4, 5).

**Table 4:** Numerical comparison between experimental and ANN ensemble mean predictions of TC for the test dataset.

TestID	Target	ANN_mean	PI_low_95	PI_High_95	AbsError	RelError_pct
1	0.39527	0.39595	0.39314	0.40285	0.00068	0.17296
2	0.39729	0.3974	0.39411	0.40222	0.00011	0.028436
3	0.40336	0.40319	0.39951	0.40545	0.00017	0.043184
4	0.40607	0.40588	0.40126	0.40852	0.00018	0.04636
5	0.41541	0.41439	0.851	0.41953	0.00012	0.029544
6	0.41957	0.41937	0.4154	0.42346	0.00019	0.047065

**Fig. 20:**
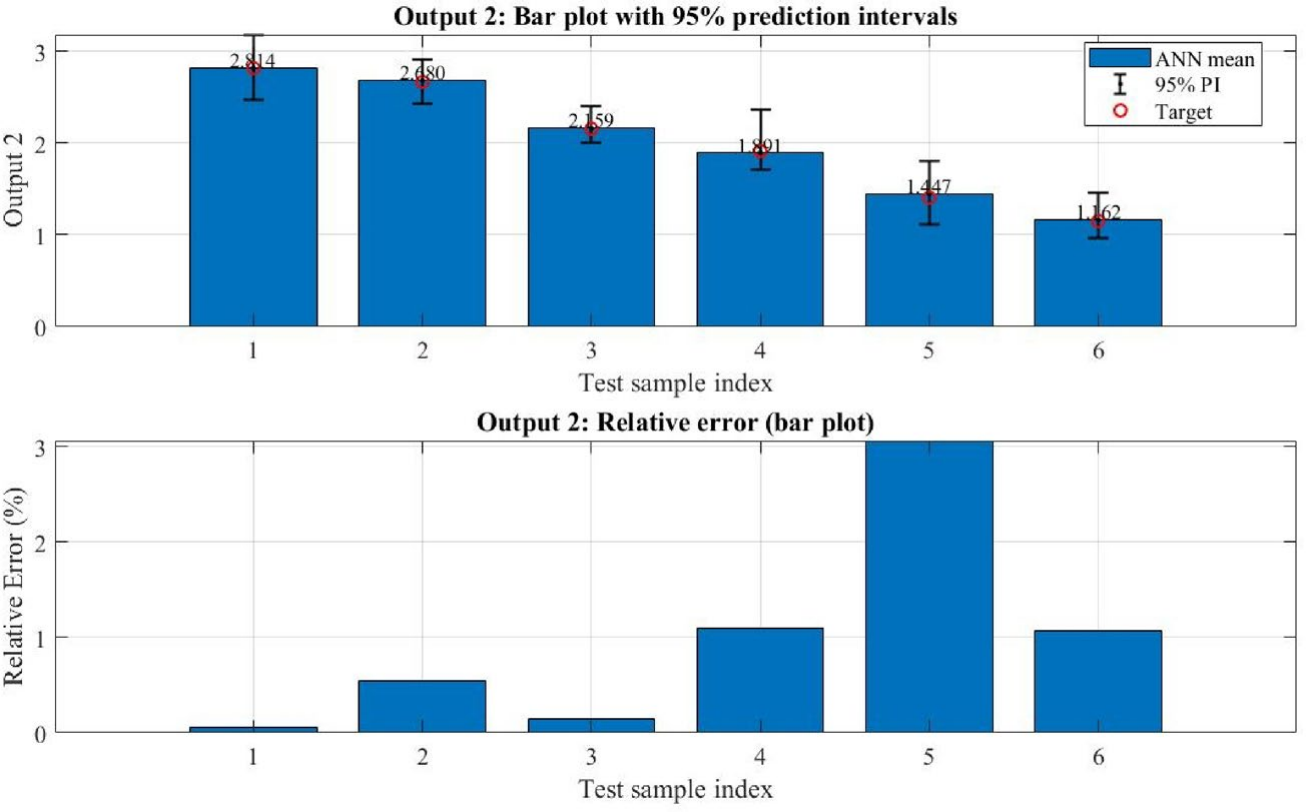
ANN ensemble mean predictions of µ_nf_ for the test dataset, including 95% prediction intervals obtained via deep ensemble modeling.

Figure 20 depicts the ANN predictions for µnf, along with the corresponding uncertainty quantification. Although the ensemble mean predictions closely matched the experimental µ_nf_ data, the prediction intervals were noticeably wider than those observed for TC, highlighting the inherently stronger nonlinear dependence of rheological behavior on T and NP-φ. Nevertheless, all experimental µ_nf_ values were fully contained within the 95% prediction intervals, demonstrating the robustness of the uncertainty propagation strategy and the reliability of the ANN framework. This behavior indicated that, despite µ_nf_’s increased sensitivity, the model provided a physically consistent and statistically reliable representation of µ_nf_ variations, which was essential for accurately evaluating flow resistance and pumping requirements in NF-based thermal systems.

**Table 5:** Numerical comparison between experimental and ANN ensemble mean predictions of µ_nf_ for the test dataset.

TestID	Target	ANN_mean	PI_low_95	PI_High_95	AbsError	RelError_pct
1	2.8155	2.8138	2.4668	3.1811	0.0016	0.059281
2	2.6651	2.6796	2.4285	2.9064	0.0144	0.54212
3	2.1556	2.1587	1.9981	2.404	0.00311	0.1447
4	1.9125	1.8915	1.7146	2.3639	0.0210	1.0988
5	1.4037	1.4467	1.1095	1.7978	0.0429	3.0614
6	1.1493	1.16.15	0.9643	1.4585	0.0122	1.066

**Fig. 21:**
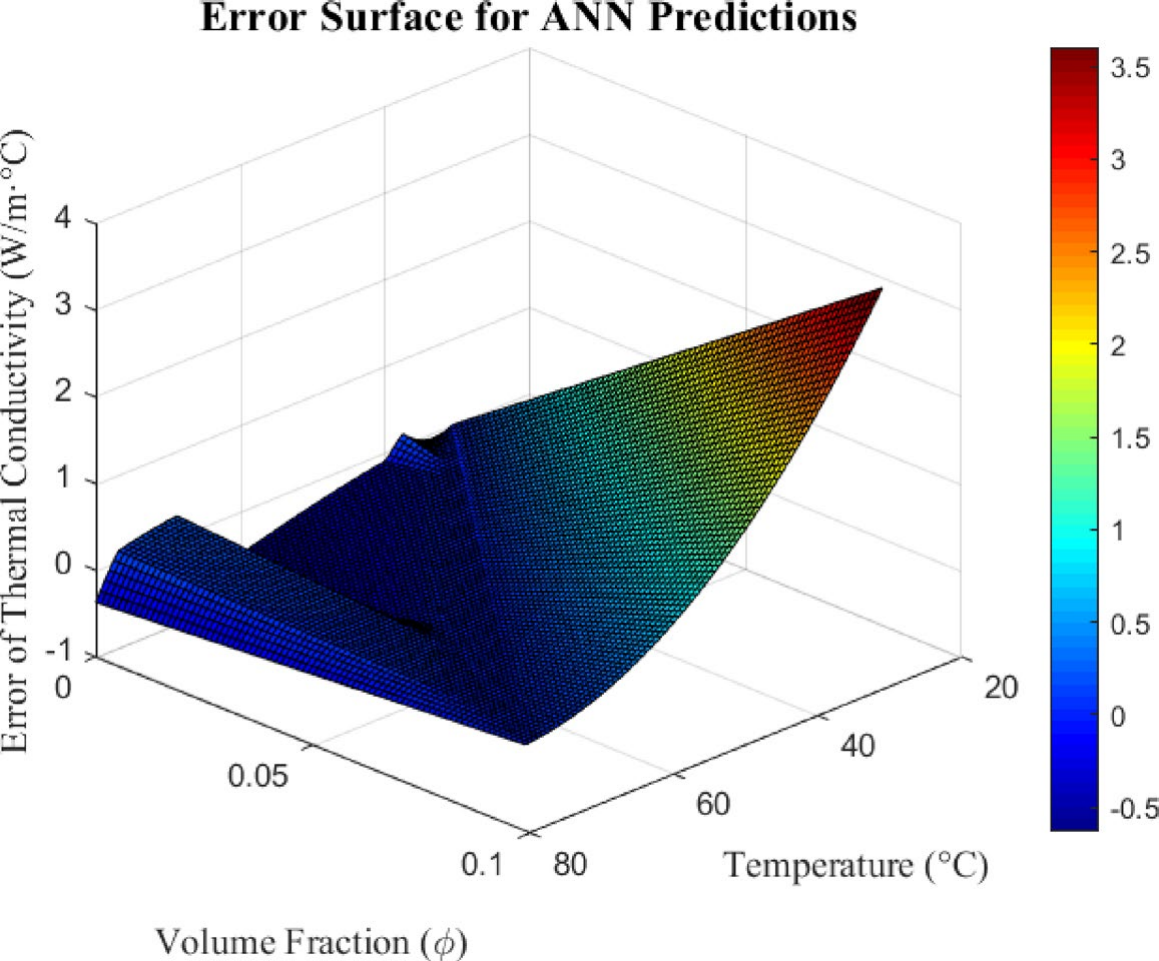
TC estimation error procedure in terms of T and NP- φ for all observational data.

Figure 21 presents the error surface of the ANN model in predicting the TC of nanocomposites under varying T and φ conditions. The results showed that the predictive framework maintains stable performance across the entire input domain, with only limited deviations from the experimental observations. Quantitatively, the minimum, maximum, and mean absolute errors were obtained as 1.3643 × 10⁻⁴ W/m·°C, 2.4464 × 10⁻² W/m·°C, and 8.9401 × 10⁻³ W/m·°C, respectively. These error levels, within narrow bounds, confirmed ANN’s ability to reliably reproduce the nonlinear dependence of TC on both thermal and compositional parameters.

**Fig. 22:**
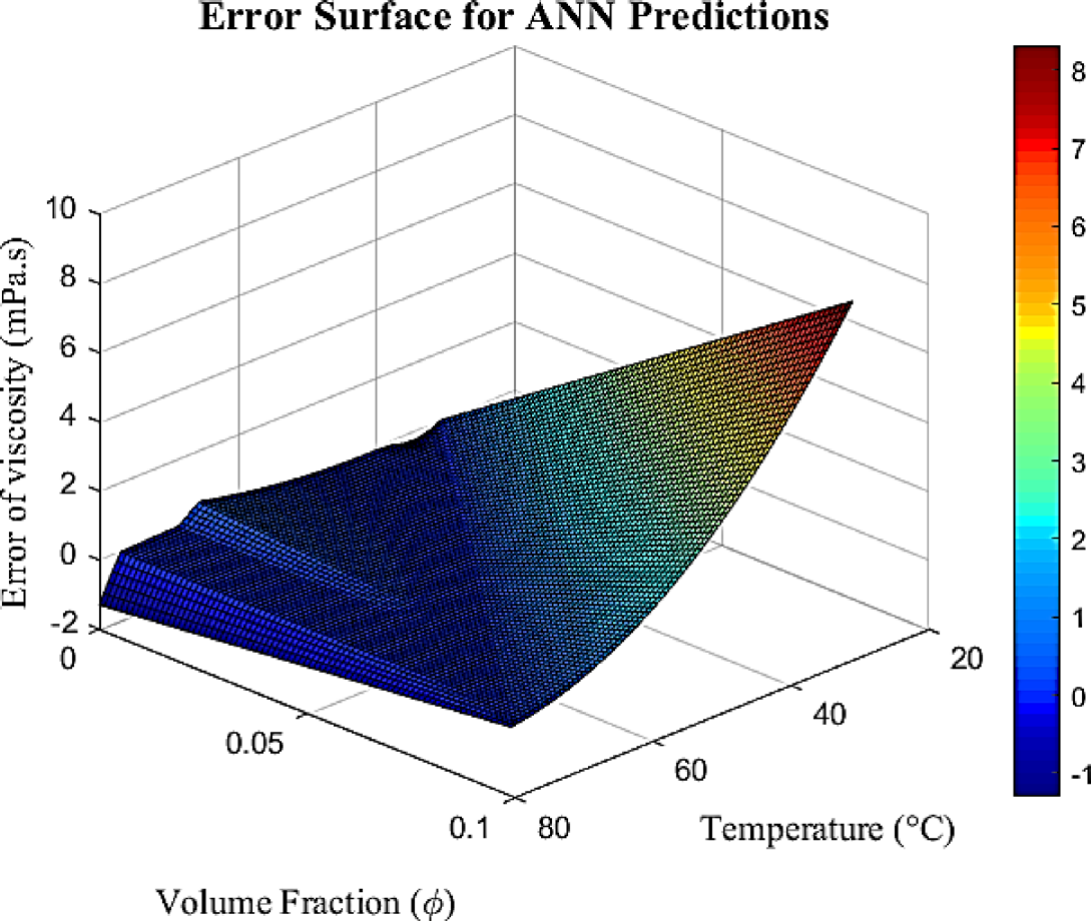
µ_nf_ estimation error procedure in terms of T and φ for all observational data.

Figure 22 illustrates the distribution of errors in the ANN predictions for µ_nf_ across the investigated ranges of T and φ. The analysis indicated that the model consistently reproduced the experimental µ_nf_ values with high fidelity, showing only minor deviations across the parameter space. The minimum, maximum, and mean absolute errors were calculated as 3.3295 × 10⁻⁴ mPa·s, 3.2540 × 10⁻² mPa·s, and 1.1972 × 10⁻² mPa·s, respectively. These bounded-error values highlighted the ANN’s strong capacity to capture the complex rheological behavior of NFs while maintaining robust generalization performance. The close alignment between predicted and experimental results further emphasized the reliability of the developed model for accurately characterizing µ_nf_ under diverse thermophysical conditions.

## Sensitivity analysis

Sensitivity analysis was performed to evaluate the robustness and interpretability of the developed ANN framework by quantifying the response of predicted TC and µ_nf_ to controlled perturbations in the input variables. In the context of thermophysical property prediction, this analysis provided insight into the relative influence of T and φ on the model outputs and assessed the stability of the predictions under realistic levels of input uncertainty. To this end, systematic variations ranging from 2% to 10% were independently applied to each input parameter, and the resulting maximum and mean percentage changes in the predicted outputs were evaluated. The consolidated results of this analysis are summarized in Table 6. The maximum deviation metrics indicated that perturbations in φ consistently induced larger variations in both TC and µ_nf_ compared with equivalent changes in T. For example, a 10% increase in T resulted in maximum deviations of 2.636% in TC and 0.623% in µ_nf_, whereas a 10% change in NP-φ led to more pronounced deviations of 5.744% and 0.893%, respectively. This behavior demonstrated greater sensitivity of thermophysical properties to NP loading and confirmed the dominant role of φ in governing the system response over the investigated range. Mean percentage changes further elucidated the overall stability of the predictive framework under moderate uncertainty conditions. Across the examined perturbation levels, average deviations in TC remained limited to 0.469%–2.001% for variations in T and 1.013%–2.186% for variations in φ. Corresponding mean deviations for µ_nf_ are even smaller, ranging from 0.096% to 0.435% under T perturbations and from 0.120% to 0.627% under φ perturbations. These results demonstrated that, despite the higher sensitivity to φ, the ANN predictions remained stable on average and exhibited only modest fluctuations across the tested uncertainty range.

**Table 6:** The Max and mean change percentage in predicted TC and µ_nf_ of Nanocomposite due to imposing 2- to 10-percent changes on each of the ANN inputs for test data.

ANN output	Max change due to φ perturbation (%)					Max change due to T perturbation (%)				
	2	4	6	8	10	2	4	6	8	10
TC (W/m·°C)	2.195	3.8	4.87	5.5	5.744	0.601	1.158	1.679	2.17	2.636
µ_nf_ (mPa.s)	0.174	0.351	0.531	0.711	0.893	0.513	1.037	1.577	2.132	2.699
ANN Output	Mean change due to NP-φ perturbation (%)					Mean change due to T perturbation (%)				
	2	4	6	8	10	2	4	6	8	10
TC (W/m·°C)	1.013	1.68	2.056	2.186	2.153	0.469	0.9	1.296	1.662	2.001
µ_nf_ (mPa.s)	0.12	0.243	0.369	0.497	0.627	0.096	0.187	0.274	0.357	0.435

ANN output	Mean change due to NP-φ perturbation (%)					Mean change due to T perturbation (%)				
	2	4	6	8	10	2	4	6	8	10
TC (W/m·°C)	1.013	1.68	2.056	2.186	2.153	0.469	0.9	1.296	1.662	2.001
µ_nf_ (mPa.s)	0.12	0.243	0.369	0.497	0.627	0.096	0.187	0.274	0.357	0.435

### Global sensitivity analysis

To overcome the inherent limitations of local one-at-a-time perturbation strategies and to explicitly account for nonlinearities and interactions among the governing input parameters, a global variance-based sensitivity analysis was conducted using Sobol’s indices. In this approach, the trained ANN surrogate model was evaluated over the full experimental ranges of T and NP-φ, allowing the total variance of the predicted thermophysical properties to be rigorously decomposed into main and interaction-driven contributions. The first-order Sobol indices indicated that T was the dominant independent contributor to the ANN prediction variance, with S1(T) = 0.5546 and a narrow 95% confidence interval of [0.5426, 0.5662]. This result demonstrated that more than half of the total output variance can be attributed solely to T variations when all other factors were averaged out. In contrast, the NP-φ showed a substantial but secondary main effect, with S1(φ) = 0.3420 and a 95% confidence interval of [0.3190, 0.3657]. These findings refined the conclusions drawn from the local sensitivity analysis by revealing that, from a global perspective, T exerted a stronger influence on the overall model response than φ. Further insight was obtained from the total-order Sobol indices, which incorporated both direct effects and higher-order interactions. The total sensitivity indices increased to ST(T) = 0.6679 and ST(φ) = 0.4578, indicating that interaction effects between T and NP loading contribute non-negligibly to the predicted response. The differences between total-order and first-order indices, namely ST(T)−S1(T) = 0.1133 and ST(φ)−S1(φ) = 0.1157, quantitatively confirmed the presence of nonlinear coupling between two input parameters. This coupling implies that the influence of each variable cannot be fully characterized in isolation, as their combined variations generate additional variance in the ANN output. The statistical robustness of the obtained sensitivity measures was verified through bootstrap resampling, yielding consistently narrow confidence intervals for all Sobol indices. This stability confirmed that the inferred ranking of parameter importance was not sensitive to sampling variability and was representative of the underlying input–output relationship captured by the ANN. Overall, while the local perturbation analysis highlighted the pronounced short-range sensitivity of predicted properties to φ, the global variance-based results showed that T remained the primary driver of output variability across the entire input space, with interaction effects playing a meaningful, quantifiable role. The combined use of local and global sensitivity analyses therefore provided a physically consistent and statistically reliable interpretation of the ANN-based predictions of thermophysical properties. From a thermophysical perspective, the stronger global contribution of T can be attributed to its simultaneous influence on multiple transport mechanisms governing MWCNT-NFs. Increasing T enhances Brownian motion and reduces intermolecular cohesive forces within the base fluid, lowering effective flow resistance and promoting interfacial energy transport between nanotubes and the surrounding fluid. These T-dependent mechanisms collectively affected both TC and µ_*nf*_ across the entire operating domain, thereby increasing the variance contribution captured by the Sobol indices. In contrast, variations in NP-φ primarily affected conductive pathway formation and particle–particle interactions, leading to more localized yet substantial changes in thermophysical behavior. The ANN framework’s ability to reproduce these coupled thermo-hydraulic trends indicated that the learned relationships remain physically meaningful and consistent with established transport mechanisms in NF systems.

## Conclusion

This study introduced a predictive framework for evaluating the thermophysical behavior of MWCNT-NFs dispersed in a H_2_O–EG (50:50) base fluid, addressing long-standing gaps in HNF research. Unlike prior studies that mainly emphasized TC, this work simultaneously considered both TC and µ_nf_, thereby offering a more balanced understanding of the competing mechanisms that govern NF performance. The developed feedforward ANN, comprising two hidden layers, demonstrated high predictive accuracy across a broad range of operating conditions, including NP-φ from 0.025% to 0.1% and Ts between 25 and 80 °C. By validating the model against experimental benchmarks, the framework demonstrated its capability to capture nonlinear trends and ensure reliable extrapolation within practical design ranges.

- Under repeated 10-fold cross-validation, the RMSE ranged from 1.31 × 10⁻⁴ to 3.71 × 10⁻⁴ W/m·°C for TC and from 0.010 to 0.031 mPa·s for dynamic µ_nf_, confirming reliable interpolation within the studied domain.
- The overall R of determination exceeded 0.99 for TC and 0.994 for µ_nf_, demonstrating strong generalization and close agreement between predicted and experimental values across the training, validation, and test datasets.
- Error analysis further confirmed the robustness of the predictions. Relative errors remained below 1.57% for TC and below 0.25% for µ_nf_, while absolute errors were tightly bound over the full input range, indicating stable model behavior.

Training convergence analysis showed early, stable optimal performance, with minimum validation errors observed at low epoch numbers for both properties. This behavior indicated efficient learning dynamics and the absence of overfitting. Sensitivity analysis revealed that NP-φ has a stronger influence than T on both properties. A 10% change in φ caused maximum deviations of 5.744% in TC and 0.893% in µ_nf_, whereas the same T variation led to smaller deviations of 2.636% and 0.623%, respectively. By jointly quantifying TC enhancement and µ_nf_ penalty, the proposed framework enabled a balanced assessment of HT gains versus flow resistance, providing practical guidance for the deployment of MWCNT NFs in advanced thermal management systems.

## Data Availability

The data that support the findings of this study are available from the corresponding author, upon reasonable request.

## Appendix

See equation (14) TC: Maxwell-type model

For TC prediction, the Maxwell effective medium model was adopted as a representative classical correlation. This model describes heat conduction in dilute suspensions of spherical inclusions dispersed in a continuous base fluid, assuming uniform particle distribution and negligible particle–particle interactions. The effective TC of the NF is given by:

14 $${k_{nf}}={k_f}=\frac{{{k_p}+2{k_f}+2\phi ({k_p} - {k_f})}}{{{k_p}+2{k_f} - \phi ({k_p} - {k_f})}}$$

Where k_nf_ is the effective TC of the NF, k_f_ denotes the TC of the base fluid, k_p_ represents the TC of the dispersed NPs, and ϕ is the NP-φ.In the present study, k_f_ was estimated from the training subset of the experimental data to avoid information leakage, while k_p_ was set to a literature-based constant representative of MWCNTs. Although originally derived for spherical particles, the Maxwell model remains a common benchmark for NF-TC predictions, particularly at low NP-φ.

See equation (15) Μnf: Brinkman/Einstein-type model

For µ_nf_ prediction, the Brinkman model, which extends the classical Einstein formulation to moderately concentrated suspensions, was employed. This correlation relates the effective µ_nf_ of the NF to the base fluid µ_nf_ and NP-φ as:

15 $${\mu _{nf}}={\mu _f}{(1 - \varphi )^{ - 2.5}}$$

Where µ_nf_ is the dynamic viscosity of the NF, µ_f_ denotes the µ_nf_ of the base fluid, and ϕ represents the NP-φ. The Brinkman model assumes Newtonian flow behavior, negligible particle aggregation, and uniform NP dispersion. Therefore, it is commonly adopted as a baseline correlation for NF-µ_nf_ prediction, including carbon-nanotube-based NFs at low to moderate φs. Consistent with the TC analysis, the base-fluid µ_nf_ was estimated exclusively from the training dataset to ensure an unbiased and consistent predictive framework.

## Abbreviations

EG: Ethylene glycol
NP: Nanoparticle
H_2_O: Water
ANN: Artificial neural networks
T: Temperature
NF: Nanofluid
TC: Thermal conductivity
µ_nf_: Viscosity
MSE: Mean squared error
R: Coefficient of determination
LM: Levenberg–Marquardt
φ: Volume concentration
MWCNTs: Multi-walled carbon nanotubes
HT: Heat transfer
